# *Ferula sinkiangensis* (Chou-AWei, Chinese *Ferula*): Traditional Uses, Phytoconstituents, Biosynthesis, and Pharmacological Activities

**DOI:** 10.3390/plants12040902

**Published:** 2023-02-16

**Authors:** Maan T. Khayat, Majed Alharbi, Kholoud F. Ghazawi, Gamal A. Mohamed, Sabrin R. M. Ibrahim

**Affiliations:** 1 Department of Pharmaceutical Chemistry, Faculty of Pharmacy, King Abdulaziz University, Jeddah 21589, Saudi Arabia; 2 Clinical Pharmacy Department, College of Pharmacy, Umm Al-Qura University, Makkah 24382, Saudi Arabia; 3 Department of Natural Products and Alternative Medicine, Faculty of Pharmacy, King Abdulaziz University, Jeddah 21589, Saudi Arabia; 4 Preparatory Year Program, Department of Chemistry, Batterjee Medical College, Jeddah 21442, Saudi Arabia; 5 Department of Pharmacognosy, Faculty of Pharmacy, Assiut University, Assiut 71526, Egypt

**Keywords:** *Ferula sinkiangensis*, Apiaceae, traditional uses, sesquiterpene coumarins, biosynthesis, bioactivities, sustainability, responsible consumption and production

## Abstract

*Ferula* is the third largest genus of the Apiaceae family, its species are utilized as a remedy for diverse ailments all over the world. *F. sinkiangensis* K. M. Shen (Chou-AWei, Chinese *Ferula*) is mainly found in Xin-jiang Uygur Autonomous Region, China. Traditionally, it is utilized for treating various illnesses such as digestive disorders, rheumatoid arthritis, wound infection, baldness, bronchitis, ovarian cysts, intestinal worms, diarrhea, malaria, abdominal mass, cold, measles, and bronchitis. It can produce different classes of metabolites such as sesquiterpene coumarins, steroidal esters, lignans, phenylpropanoids, sesquiterpenes, monoterpenes, coumarins, organic acid glycosides, and sulfur-containing compounds with prominent bioactivities. The objective of this work is to point out the reported data on *F. sinkiangensis,* including traditional uses, phytoconstituents, biosynthesis, and bioactivities. In the current work, 194 metabolites were reported from *F. sinkiangensis* in the period from 1987 to the end of 2022. Nevertheless, future work should be directed to conduct *in vivo*, mechanistic, and clinical assessments of this plant`s metabolites to confirm its safe usage.

## 1. Introduction

People have utilized plants since ancient times for different reasons: food, clothing, shelter, decoration, and construction [1]. Their usage by local and indigenous communities has been vertically and orally transferred among generations [2]. Also, plants are dynamic factories for the production of enormous kinds of metabolites. The plants and/or their metabolites form the backbone for diverse pharmaceutics, perfume, cosmetic, agrochemical, and food industries. Besides, they are traditional remedies for many ailments in various countries particularly the developed ones [3,4].

*Ferula* is the third largest genus of Apiaceae family that comprises about 180 species. Its species commonly exist in Asian and Mediterranean regions e.g., Iran, Turkey, Algeria, Afghanistan, Saudi Arabia, Pakistan, China, and India [5]. *Ferula* means “carrier” or “vehicle” in Latin and this genus is distinguished by the existence of oleo-gum-resins (e.g., sagapenum, asafoetida, ammoniacum, and galbanum) [6]. Most of its plants are with a pungent odor and bitter taste due to the existence of disulfides [7]. In Asia, they are utilized as a spice and in pickles, meat sauces, curry, and other foods as flavoring agents [7]. In China, the *Ferula* resin is employed for treating dysentery, worms, and malaria, and to dissolve phlegm, as well as an insecticide and deodorant [8]. Its plants are utilized as tranquilizers and for treating rheumatism, digestive disorders, headache, dizziness, toothache, and arthritis [9]. Also, they are of great significance in traditional and folk medicine for more than thousand years in treating epilepsy, asthma, stomachache and headache, intestinal parasites, flatulence, influenza, dysentery, and weak digestion in different countries [10]. These plants displayed a myriad of bioactivities: anticancer, anthelmintic, antiepileptic, antioxidant, antiulcer, antimicrobial, antihypertensive, antifungal, antidepressant, antiproliferative, antiprotozoal, antihemolytic, antimycobacterial, anticoagulant, antifertility, antispasmodic, anticonvulsant, relaxant, antinociceptive, hypnotic, memory and digestive enzyme enhancing, antiviral, anxiolytics, antihyperlipidemic, antigenotoxic, anti-inflammatory, antihyperglycemic, antidiabetic, and hepatoprotective [11,12,13]. They also demonstrated aphicidal, phytotoxic, and acaricidal activities [11,12,13]. It was stated that sesquiterpene coumarins, coumarins, aromatic acid lactones, and sesquiterpenes are the prime phytoconstituents of *Ferula* plants roots [11,14], while sesquiterpenes and monoterpenes and their oxygenated derivatives with diversified structures are the principal metabolites of *Ferula* species aerial parts oil [15].

It is worth reporting that the improper practice of wild plants is commonly invasive and devasting to the naturally existing medicinal plants which may cause a dangerous menace to these substantial plants and may result in the extinction of some valuable species [16]. Thus, the conservation of land resources and responsible consumption and production are the challenges in sustainable land resources usage [16].

**Figure 1:**
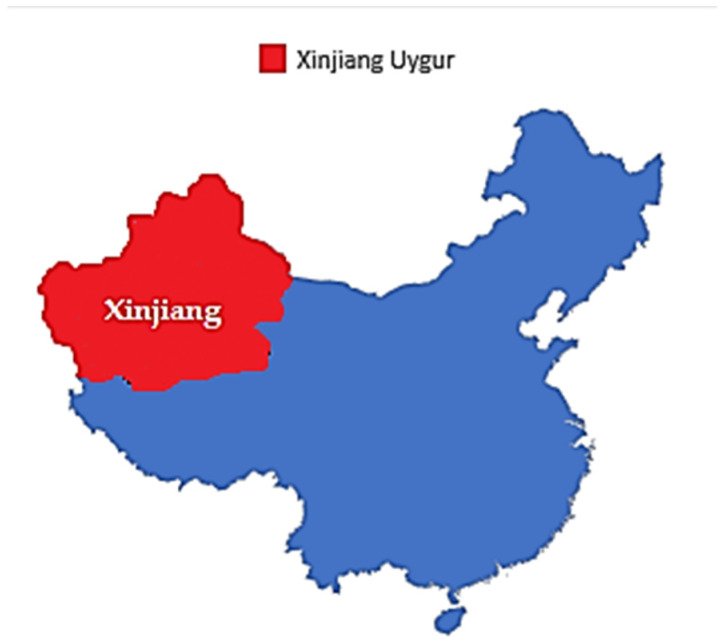
A map illustrating the geographic location of the Xinjiang-Uigur Autonomous Region in China.

*F. sinkiangensis* K.M. Shen (Chou-AWei, Chinese *Ferula*, (Xinjiang’awei)) is an important member of this genus. *F. sinkiangensis* is a perennial plant endemic in Xinjiang Uygur Autonomous Region, China [17] (Figure 1).

It was reported that this plant is in the menace of evanescence due to irrigation, road building, unconstrained mining, reclamation, climate variation, and original habitat deterioration, leading to annual shrinkage of *F. sinkiangensis* resources [18]. This plant was included among 2nd class protected wild medicinal species and 3rd class endangered plant in China [19,20]. However, this plant has not yet been included in the IUCN Red List of threatened species, as well as in CITES (Convention on International Trade in Endangered Species of Wild Fauna and Flora) [21,22,23].

Additionally, various metabolites have been identified from its roots, oleo-gum-resin, resin, seeds, and aerial parts such as steroidal esters, phenylpropanoids, sesquiterpene coumarins, aromatic acids, sesquiterpenes, coumarins, monoterpenes, lignans, and sulfanes [11,14,24,25,26,27,28,29,30,31].

The plant and its phytoconstituents revealed various bioactivities such as anti-ulcerative, antibacterial, anti-inflammatory, antioxidant, molluscicidal, anti-schistosome, anti-drug addiction, immunopharmacological, anti-neuroinflammatory, anticancer, antifungal, antiviral, and insecticidal [11,14,25,26,27,28,29,30,31,32]. It is noteworthy that there is no current inclusive review on this plant. Since 1987, many studies were performed revealing new metabolites with diverse structural variation and promising activities from this plant. In this work, the reported studies on this plant, including traditional uses, its metabolites, their structural classes, biosynthesis, and bioactivities are reviewed. Overall, this work intended to give an inclusive introduction to *F. sinkiangensis* that could help in identifying the future investigations direction and possible implementations of this valuable medicinal plant.

## 2. Research Methodology

To collect the reported data on *F. sinkiangensis*, a comprehensive search was carried out on PubMed (37 records) and Google-scholar (529 records) databases, as well as the published articles by various publishers, including Springer, Elsevier, Taylor & Francis, Wiley, MDPI, Thieme, Hindawi, etc. The search keywords were *F. sinkiangensis*, ethnomedicinal uses, folk uses, bioactive compounds, biosynthesis, phytochemistry, biological activity, and pharmacology.

**Figure 2:**
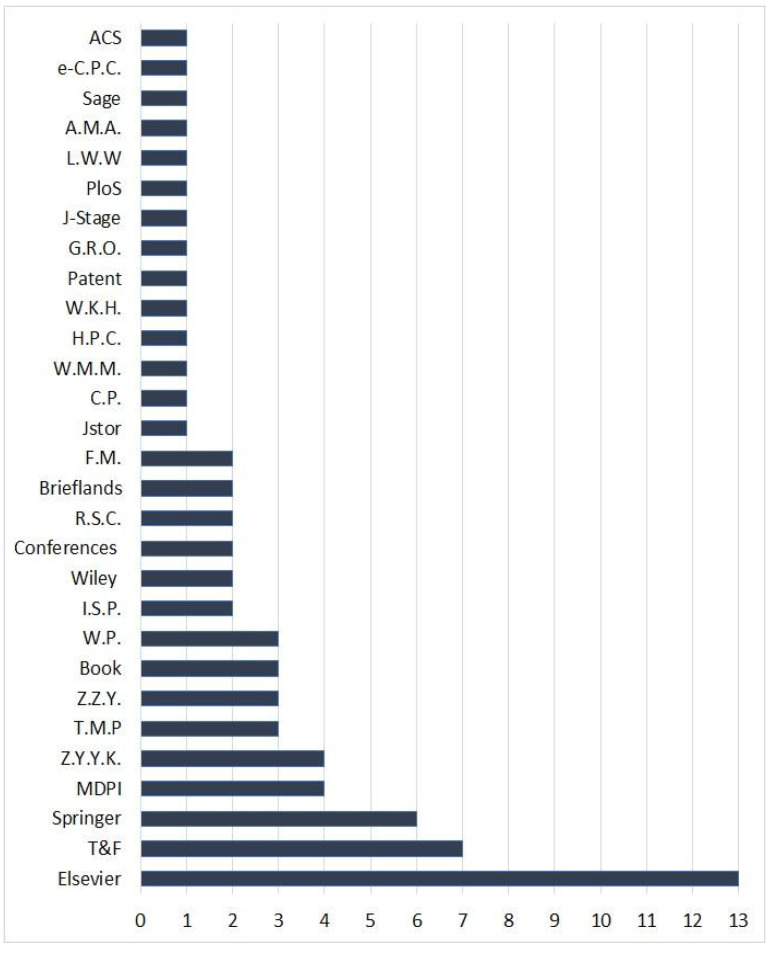
Sources of the cited references in this work and their numbers. T&F: Taylor and Francis Ltd.; MDPI: Multidisciplinary Digital Publishing Institute; Z.Y.Y.K.: Zhongguo Yufang Yixue Kexueyuan; T.M.P.: Thieme Medical Publishers; Z.Z.Y.: Zhongguo Zhongyi Yanjiuyuan; W.P.: Webpages; I.S.P: Iranian Society of Pharmacognosy; R.S.C.: Royal Society of Chemistry; F.M.: Frontiers Media; C.P.: Cell Press; W.M.M.: Walsh Medical Media; H.P.C.: Hindawi Publishing Corporation; W.K.H.: Wolters Kluwer Health—Pvt.; G.R.O.: Global Research Online; J-Stage: Pharmaceutical Society of Japan; PLOS: Public Library of Science; L.W.W.: Lippincott Williams and Wilkins Ltd.; A.M.A.: American Medical Association; e-C.P.C.: e-Century Publishing Corporation; A.C.S. American Chemical Society.

The selection criteria of the records including in this work were: (1) research articles had to be published in scientific journals (2) studies that reported the traditional uses, metabolites, biosynthesis, and bioactivities of *F. sinkiangensis* (3) patents, book chapters, and conferences. The covered records in this work included the published articles from various publishers, patents, book chapters, and conferences in the period from 1987 to the end of 2022. For the non-English articles, English abstracts have been utilized. The studies that did not agree with the selection criteria, as well as the whole non-English, non-reviewed, and not journal articles are excluded. In the current work, 72 references have been cited including articles from various publishers, books, conferences, webpages, and patents (Figure 2).

## 3. Taxonomy of *F. sinkiangensis* [32]

KingdomPlantaeDivisionMagnoliophytaClassMagnoliopsidaFamilyApiaceaeGenus
*Ferula*
Species
*sinkiangensis*


## 4. Traditional Uses of *F. sinkiangensis*

*F. sinkiangensis* is mainly found in Xinjiang, which is a region with various minorities. The plant has been described in the Chinese Pharmacopoeia and in “Medica of the Tang Dynasty” for a long time as a folk medicine for gastric disorders and rheumatoid arthritis [24].

The resin of the roots or stems of *F. sinkiangensis* (Ferulae Resina, “AWei” in China) is a folk medicine recorded in Chinese Pharmacopoeia [25]. It is often utilized for reducing the symptoms of lumps, indigestion, joint pain, wound infection, baldness, bronchitis, and ovarian cysts by Uygur people in Xinjiang [25,33,34,35]. It also is efficient in killing intestinal worms, as well as treating parasite-caused malnutrition, abdominal and stomachic swelling pain, diarrhea, malaria, abdominal mass, cold, and measles. However, its powerful odor has restricted its usage [36,37]. The resin is indicated in treating animal stagnation and food accumulation, concertions and conglomerations because of blood stasis, abdominal syndrome, and abdominal pain due to accumulation of worms, also for malaria and dysentery [38] at doses 1–1.5 g in the form of pills or oral powder. The resin should not be decocted with H_2_O [38]. Its use is prohibited for patients with spleen and stomach weakness, as well as for pregnant women [36,38].

## 5. Phytoconstituents of *F. sinkiangensis*

The phytochemical investigation of different parts of *F. sinkiangensis*, including gum resin, aerial parts, seed, roots, oleo-gum-resin, and resins led to the separation of different classes of phytoconstituents by the mean of diverse chromatographic tools (Table S1). Their structure characterization was performed using various spectral techniques (e.g., UV, NMR, MS), as well as CD, [α]_D_, and Xray analyses and chemical means. A total of 194 metabolites were separated from *F. sinkiangensis* (excluding polysaccharides). These metabolites were highlighted below.

### 5.1. Sesquiterpene Coumarins

**Figure 3:**
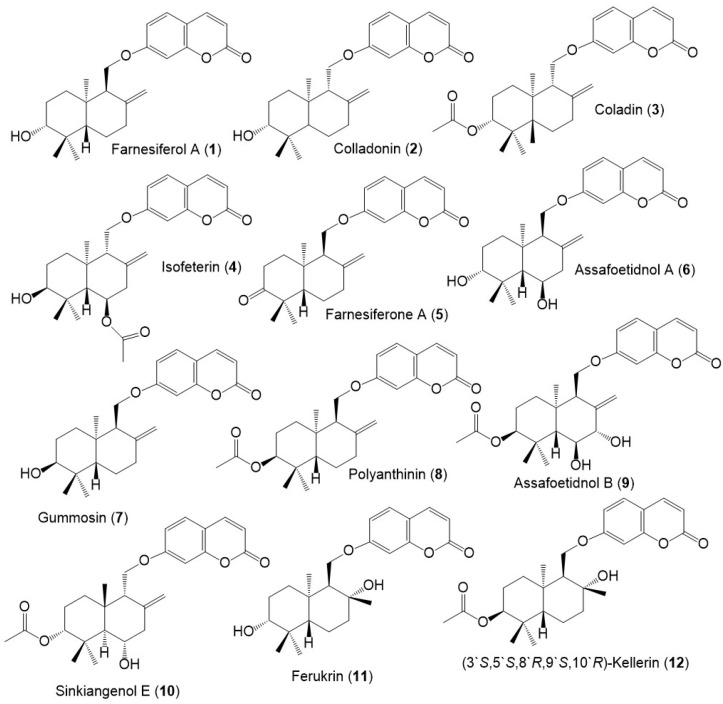
Structures of sesquiterpene coumarins (**1**–**12**) reported from *F. sinkiangensis*.

**Figure 4:**
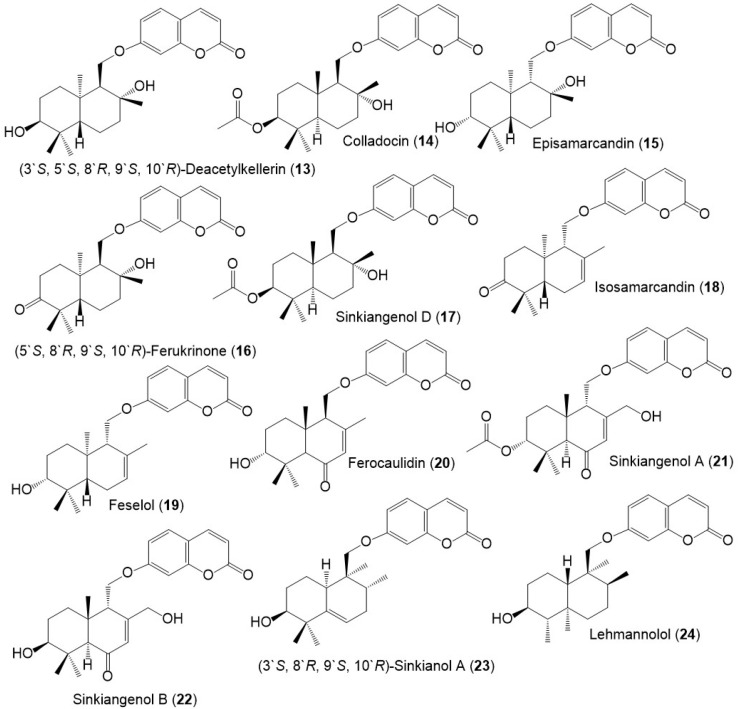
Structures of sesquiterpene coumarins (**13**–**24**) reported from *F. sinkiangensis*.

**Figure 5:**
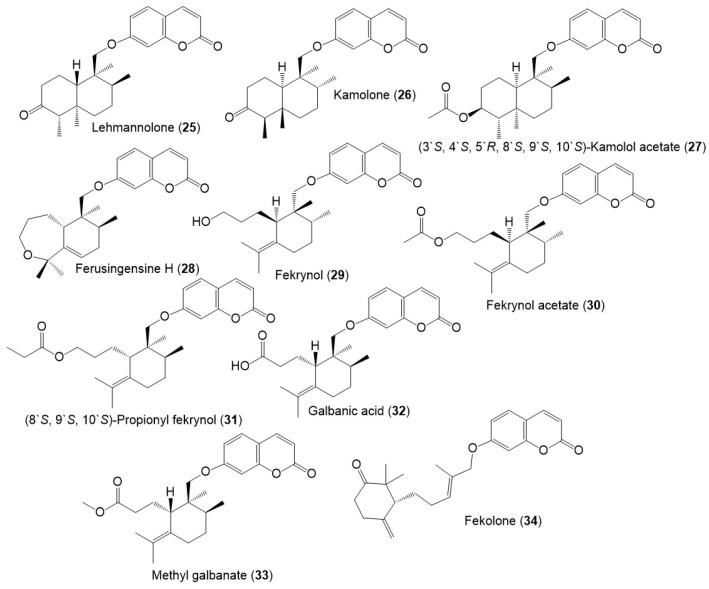
Structures of sesquiterpene coumarins (**25**–**34**) reported from *F. sinkiangensis*.

**Figure 6:**
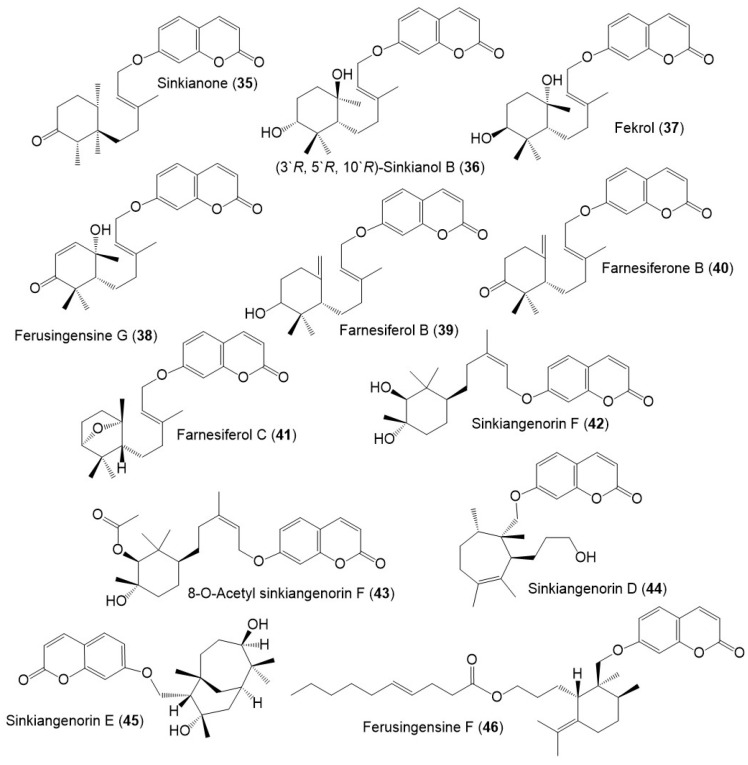
Structures of sesquiterpene coumarins (**35**–**46**) reported from *F. sinkiangensis*.

**Figure 7:**
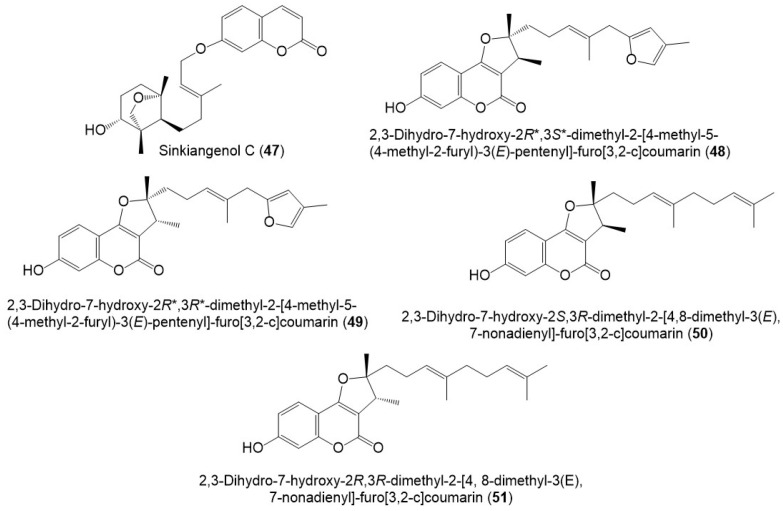
Structures of sesquiterpene coumarins (**47**–**51**) reported from *F. sinkiangensis*.

**Figure 8:**
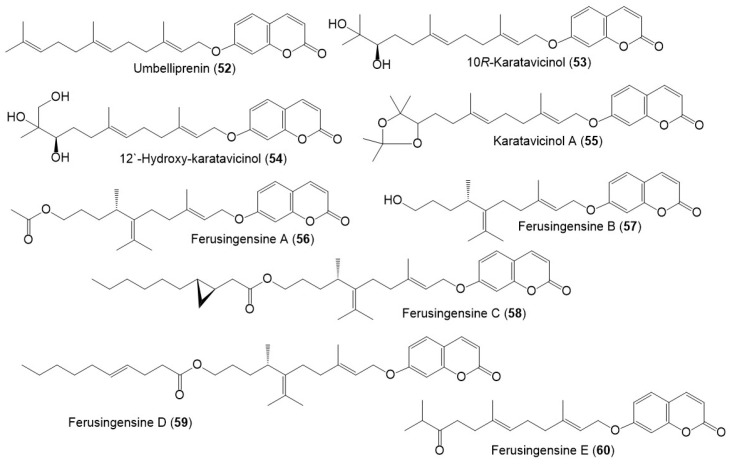
Structures of sesquiterpene coumarins (**52**–**60**) reported from *F. sinkiangensis*.

The reported studies showed that sesquiterpene coumarins represent the major metabolites produced by this plant. They represent 60 metabolites (Figure 3, Figure 4, Figure 5, Figure 6, Figure 7 and Figure 8) of the total compounds reported from this plant that were mainly separated from gum resin, seed, roots, and resins. It was noted that no sesquiterpene coumarin derivatives were reported from the aerial parts.

These compounds featured linked coumarin and sesquiterpene units through C–O–C ether bridge. These metabolites include monocyclic, bicyclic, or chain derivatives. Also, they could be accountable for many of the stated bioactivities of this plant.

Their separation was performed by different chromatographic techniques, including SiO_2_/RP-18/Sephadex LH-20/HPLC, whereas the identification and configuration were accomplished using assort spectral tools (e.g., UV, NMR, MS), as well as CD, [α]_D_ and Xray analyses. They had UV absorbance at 320–330 nm and a common fragment at *m*/*z* 185 in MS [39].

Among these metabolites, karatavicinol A (**55**), a new sesquiterpene coumarin along with **32**, **39**, **41**, **52**, and **53** were purified from the antiulcer resin CHCl_3_ extract [14] (Figure 5).

### 5.2. Sesquiterpene Chromones and Monoterpene Coumarins

**Scheme 1:**
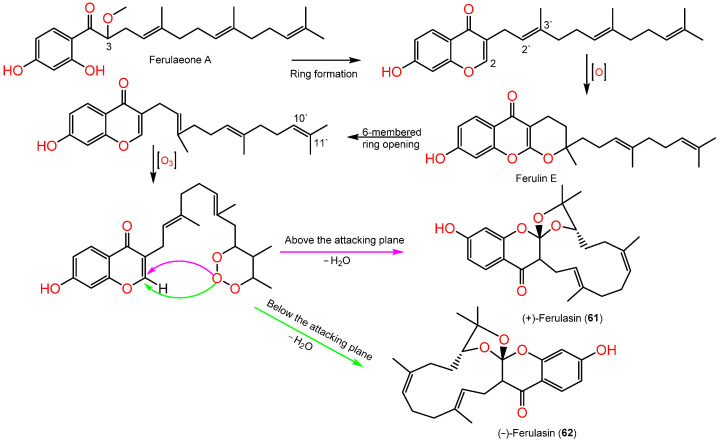
Biosynthetic pathway of (±)-ferulasin [40].

**Figure 9:**
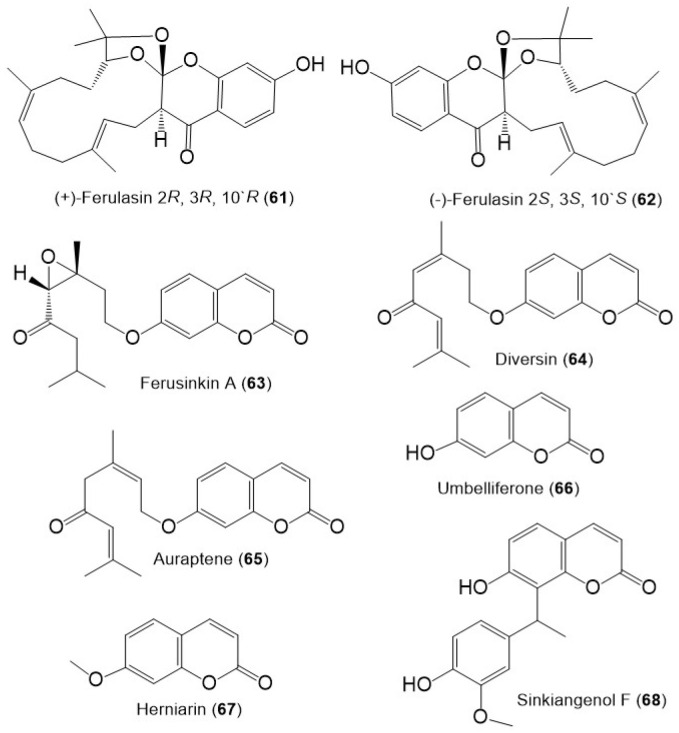
Structures of sesquiterpene chromones (**61** and **62**), monoterpene coumarins (**63**–**65**), and coumarins (**66**–**68**) from *F. sinkiangensis*.

Sesquiterpene chromones possessing a 24-carbon skeleton consisting of sesquiterpene and chromone were reported from *F. sinkiangensis* roots. In 2022, Wang et al., reported the purification of new derivatives, (±)-ferulasin from the roots MeOH extract that was established by diverse spectral, Xray, and ECD analyses. Ferulasins (**61** and **62**) showed an unusual oxygen-bearing macrocyclic skeleton with a tri-oxaspiro unit and a new backbone in which the C-10` and C-11` of the sesquiterpene side chain form an oxygen-including 13-membered ring with C-2 of chromone (Figure 9). It was obtained as an enantiomeric mixture that was chiral-separated by HPLC to (+)-**61** and (-)-**62** with 2R/3R/10`R and 2S/3S/10`S configurations, respectively based on Xray and ECD data [40]. Wang et al., assumed the biosynthesis of **61** and **62** from ferulaeone A (**71**). The reduction of the **71**-side chain C-3 produces 6-membered ring containing oxygen (Scheme 1). After that, the side chain C2`-C3` is oxidized and yields a 6-membered ring having oxygen with C-2 of chromone. After the six-membered ring fission, C10`-C11` bond reacts with ozone. Lastly, attack of oxygen-atoms to C2-C3 the double bond from below or above the plane with removal of H_2_O a molecule to afford **61** and **62** (Scheme 1).

Additionally, ferusinkin A (**63**), a rare new monoterpene coumarin and known analogs **64** and **65** were purified and identified by Liu et al. in 2020 from the aerial parts MeOH extract (Figure 9) [41].

### 5.3. Coumarins

The coumarins; **66** and **67** were purified from the *F. sinkiangensis* aerial parts and characterized based on spectral and physical data [41]. Additionally, sinkiangenol F (**68**) a new coumarin was purified from the resin EtOH extract. This compound is rare coumarin derivative having a coumarin unit connected to phenylethane moiety by C–C linkage at C-8 [39].

### 5.4. Sesquiterpene Phenylpropanoids

**Figure 10:**
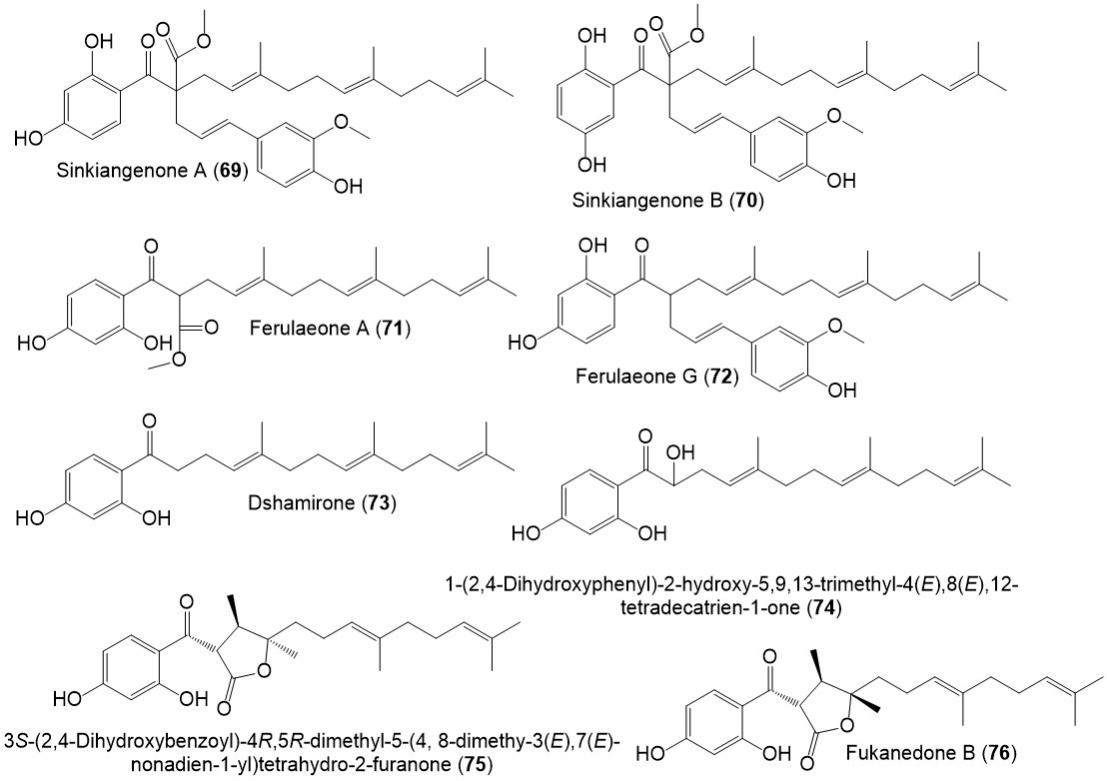
Structures of sesquiterpene phenylpropanoids (**69**–**76**) from *F. sinkiangensis*.

Sesquiterpene phenylpropanoid derivatives were commonly separated from *Ferula* genus [42]. In 2018, Wang et al., stated the separation of new sesquiterpene phenylpropanoids, sinkiangenones A (**69**), and B (**70**), along with **72** from the resin 95% EtOH extract, which were specified utilizing spectral and CD analyses (Figure 10) [29].

Also, Wang et al., separated sesquiterpene phenylpropanoid derivatives (**71** and **73**–**76**) from *F. sinkiangensis* roots MeOH extract [43]. It is noteworthy that these derivatives were previously reported from other *Ferula* species: **71** from *F. ferulioides* roots; **73** from the underground parts of *F. heuffelii* and roots of *F. ferulioides*, *F. fukanensis*, *F. dubjanskyi*, and *F. mongolica*; **74** from *F. ferulioides* roots; **75** from *F. heuffelii*, *F. fukanensis*, and *F. ferulioides* roots, and **76** from *F. heuffelii* and *F. ferulioides* roots [42,43], suggesting the close chemotaxonomic relation of *F. sinkiangensis* and the other *Ferula* species, therefore, they could share the biosynthetic pathways of these metabolites [43].

### 5.5. Lignans

**Figure 11:**
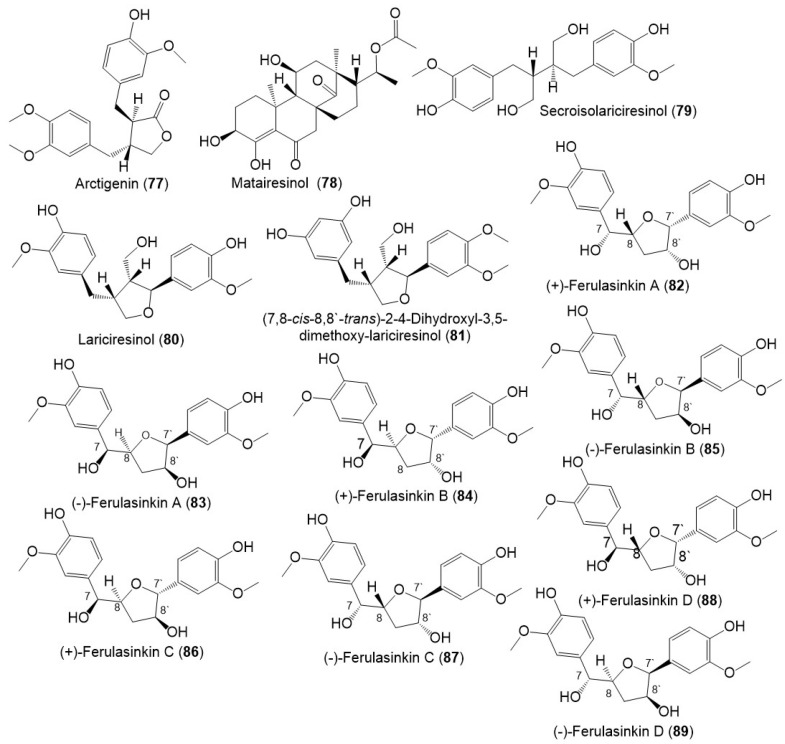
Structures of lignans (**77**–**89**) reported from *F. sinkiangensis*.

Lignans, norlignans, and sesquilignans were reported mainly from *F. sinkiangensis* seeds and resins. (±)-Ferulasinkins A–D (**82**–**89**) (Figure 11), new norlignans characterized by tetrahydrofuran rings were separated as racemic mixtures from the EtOAc fraction of the resins 95% EtOH extract by MCI gel CHP 20P/RP-18/YMC gel ODS-A-HG/SiO_2_/Sephadex LH-20/preparative TLC.

The chiral column HPLC separation afforded their (*-*)- and (+)-antipodes. Their structures and configurations were specified by spectral tools and computational methods [28].

**Figure 12:**
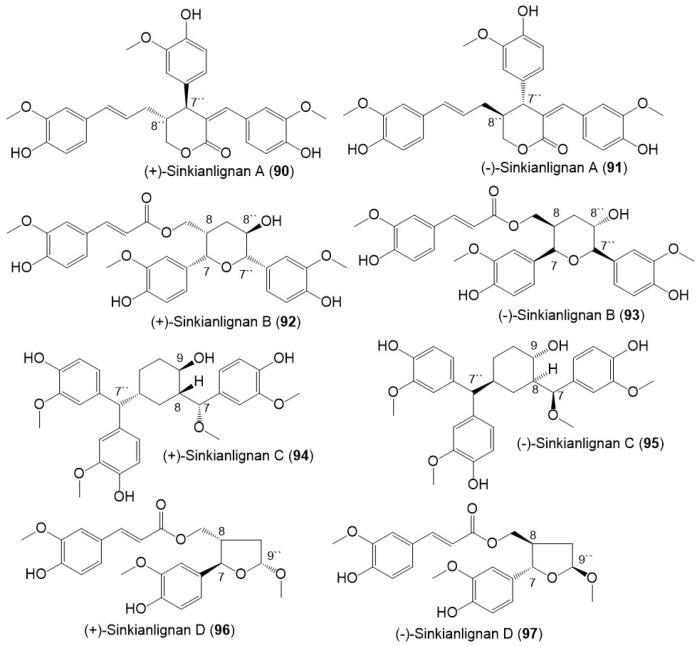
Structures of lignans (**90**–**97**) reported from *F. sinkiangensis*.

**Figure 13:**
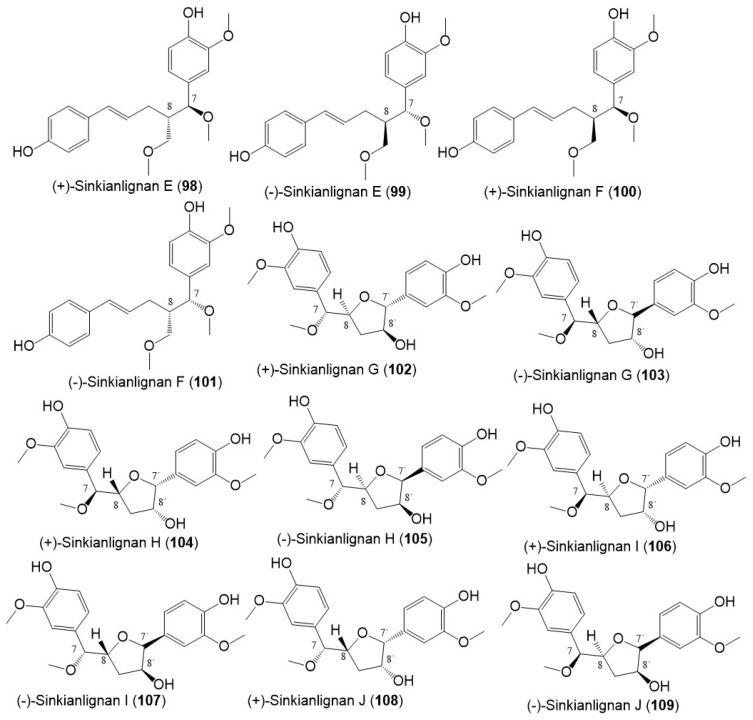
Structures of lignans (**98**–**109**) reported from *F. sinkiangensis*.

Additionally, Li et al., purified new racemic sesquilignans; sinkianlignans (±)-A–D (**90**–**97**) characterized by a rare α-γ′, β-γ′, and γ-γ′ linkage pattern, and new lignans; sinkianlignans (±)-E–F (**98**–**101**) from the resin 95% EtOH extract using SiO_2_/RP-18/MCI gel CHP 20P/YMC gel ODS-A-HG/Sephadex LH-20 CC/preparative TLC and chiral HPLC and elucidated by spectral and computational tools (Figure 12 and Figure 13) [44].

**Scheme 2:**
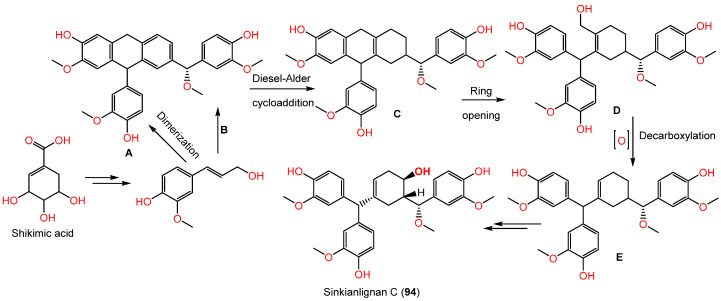
Biosynthesis of sinkianlignan C (**94**) via shikimate pathway [44].

Sesquilignans are type of lignans that consist of 3 phenylpropanoid units. Compound **94** was assumed to be biosynthesized by the shikimate pathway (Scheme 2). First, phenylpropanoid is formed by a shikimic acid pathway that undergoes polymerization to produce intermediate A (aryltetralin lignan). In addition, intermediate C with a new six-membered ring skeleton is yielded from the intermediates **A** and **B** by the Diesel-Alder cycloaddition reaction. Moreover, **C** produces **D** by opening the ring at C1−C7. Subsequent oxidization and decarboxylation of **D** yields **E**. After a set of redox reactions, intermediate **E** gives **94** [44].

**Figure 14:**
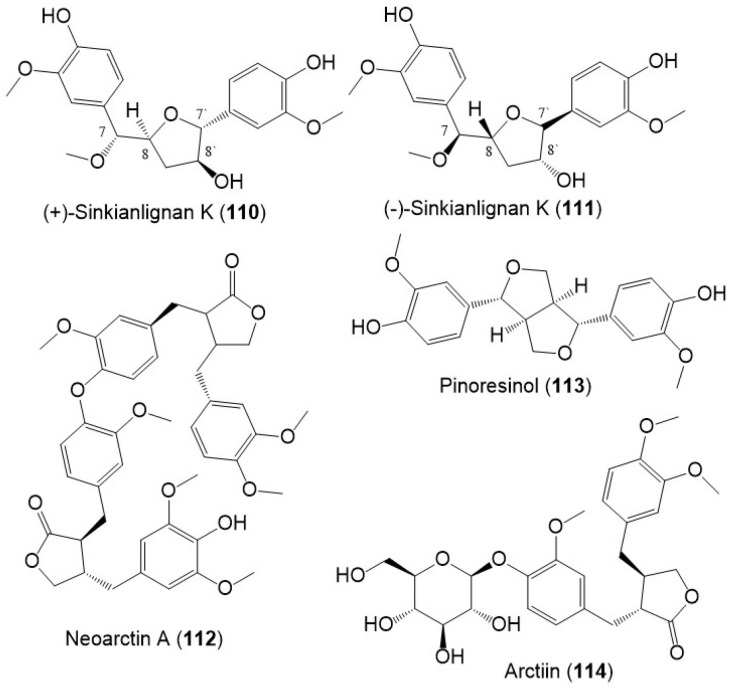
Structures of lignans (**110**–**114**) reported from *F. sinkiangensis*.

Sinkianlignans G–K (**102**–**111**) new norneolignans were purified from 95% resin EtOH extract utilizing SiO_2_/RP-18/MCI gel CHP 20P/Sephadex LH-20/preparative TLC (Figure 14). Compounds **102**–**111** were obtained as racemic mixtures that were separated by chiral HPLC and characterized by spectral and computational means [18].

### 5.6. Sesquiterpenes and Monoterpenes

**Figure 15:**
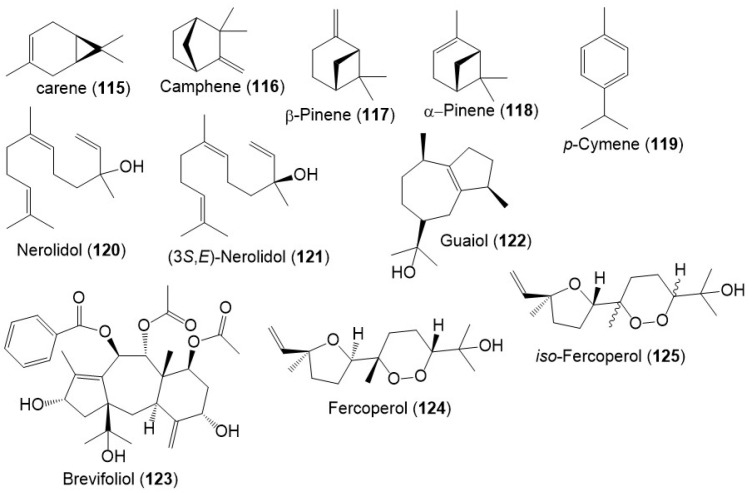
Structures of monoterpenes (**115**–**119**) and sesquiterpenes (**120**–**125**) reported from *F. sinkiangensis*.

Besides, monoterpenes (e.g., carene (**115)**, camphene (**116)**, β-pinene (**117),** α- pinene (**118)**, and p-cymene (**119)**) were encountered in the *F. sinkiangensis* oleo-gum resins` volatile oil (Figure 15) [8]. Further, Wang et al., purified a new sesquiterpenoid **125**, along with **120**, **122**, and **124** from *F. sinkiangensis* roots. Compounds **124** and **125** were isomeric cyclic-endoperoxy-nerlildol sesquiterpene derivatives, whereas **120** and **122** were chain and guaiane-type sesquiterpenoid, respectively [40].

### 5.7. Sulfanes

**Figure 16:**
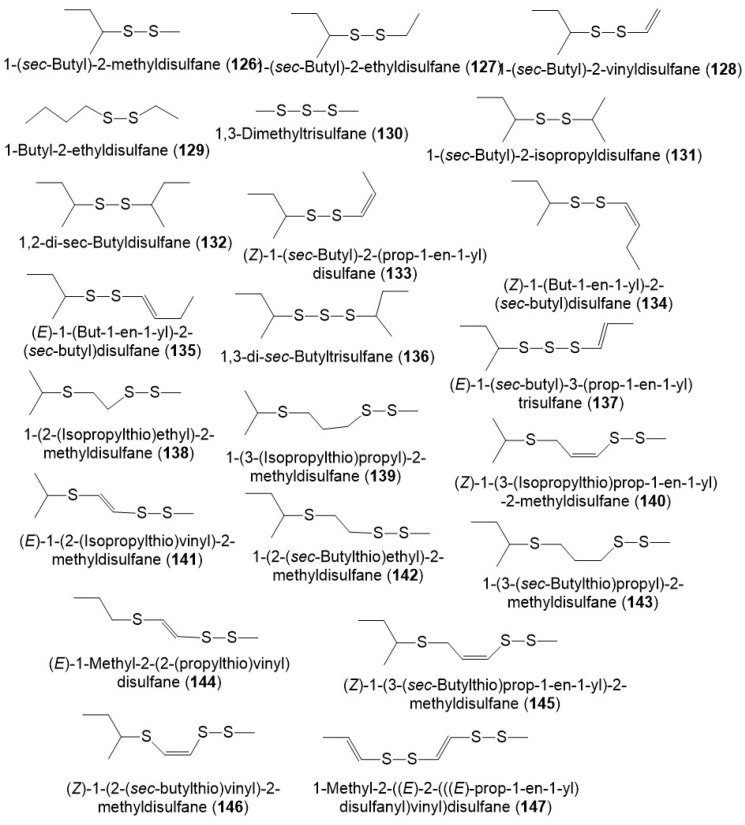
Structures of sulfanes (**126**–**147**) reported from *F. sinkiangensis*.

It was reported that polysulfides including disulfanes, trisulfanes, di-disulfanes, and thio-disulfanes are the predominant constituents of the *F. sinkiangensis* volatile oil oleo-gum resins. The oil content was 16.7% of which 64.1% were sulfur compounds. The disulfanes were the prime components: **126**–**130**, **134**, and **135** (Figure 16) [8]. Further, the GC-MS analysis of essential oil (3.8% yield) of *F. sikiangensis* seeds obtained from Xinjiang, China that was prepared by hydro-distillation method revealed the existence of 26 metabolites, comprising 99.001% of total oil [33].

### 5.8. Sterols

**Figure 17:**
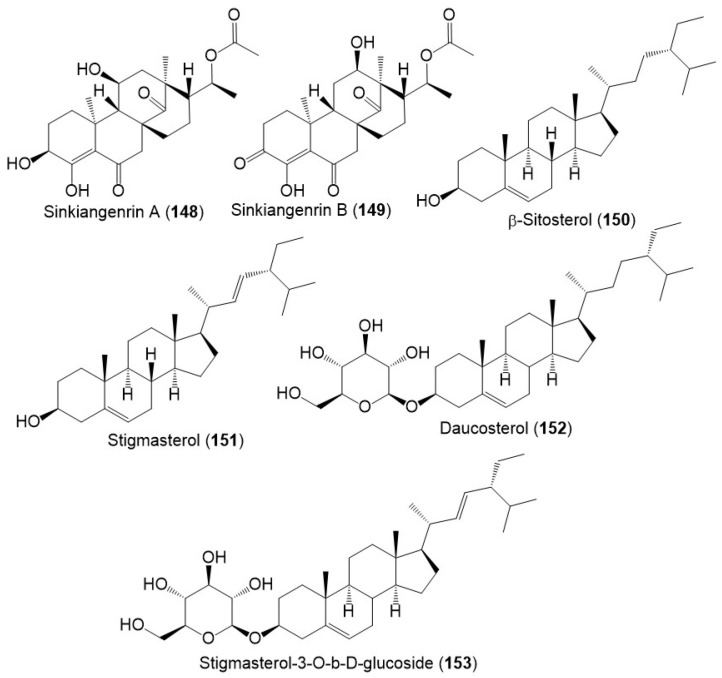
Structures of sterols (**148**–**153**) reported from *F. sinkiangensis*.

Chromatography separation of *F. sinkiangensis* 95% EtOH seed extract using SiO_2_, Sephadex LH-20, and HPLC yielded new steroidal esters: sinkiangenrins A (**148**) and B (**149**) that were characterized by NMR and Xray analyses [17]. These compounds are related to oleagenin-cardenolide with different C-13/C-10/C-9/C-8 configuration, having 3S/8R/9S/10S/11S/13S/17R/18R and 8S/9S/10S/12R/13R/17R/18R-configurations, respectively (Figure 17).

**Scheme 3:**
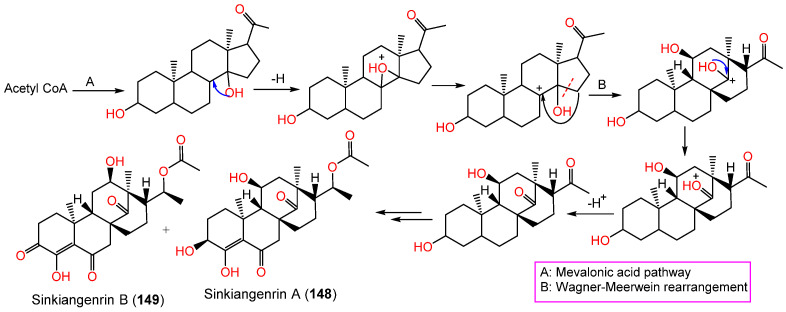
Biosynthesis of sinkiangenrins A (**148**) and B (**149**) [17].

These metabolites have an unparallel carbon framework that originates from C21-steroids (Scheme 3). Firstly, the initiation of D-ring rearrangement by C8–C14 pregnane epoxide formation, then C-8 carbocation formation. After that, Wagner–Meerwein rearrangement results in a C14–15 bond migration to C-8 and the creation of a C-14 protonated carbonyl that is deprotonated [45]. Following that set of enzyme-catalyzed reactions produce **148** and **149** [17].

### 5.9. Phenolic Compounds and Other Metabolites

**Figure 18:**
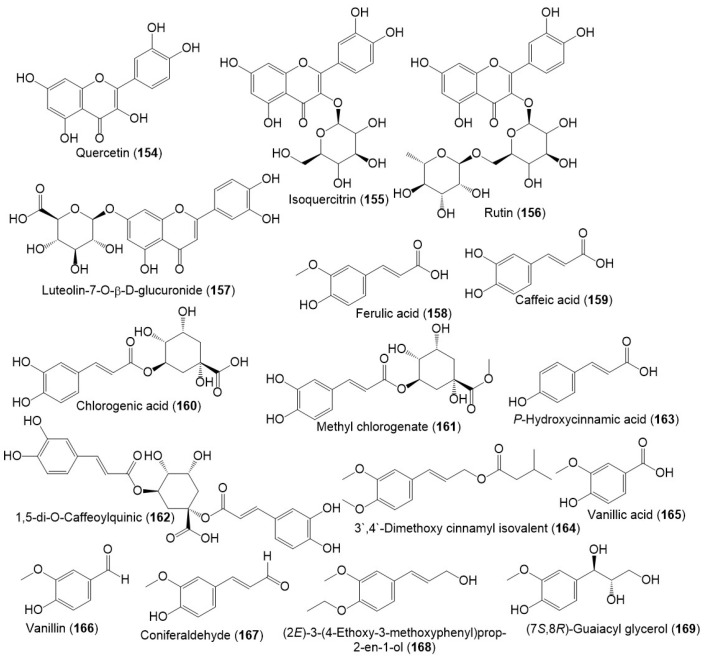
Structures of phenolic compounds (**154**–**169**) reported from *F. sinkiangensis*.

**Figure 19:**
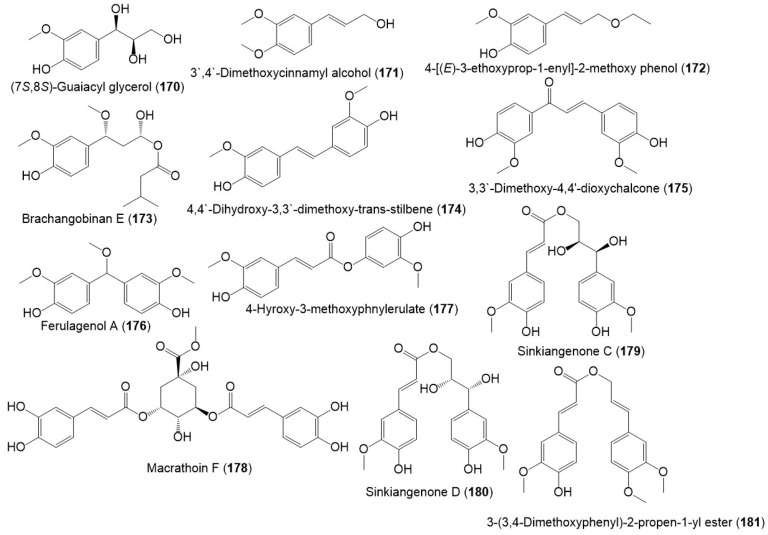
Structures of phenolic compounds (**170**–**181**) reported from *F. sinkiangensis*.

Several studies reported the separation of phenolics metabolites such as flavonoids, phenylpropanoids, and acids from resin and seed extracts (Figure 18 and Figure 19) [18,24,27,29].

**Figure 20:**
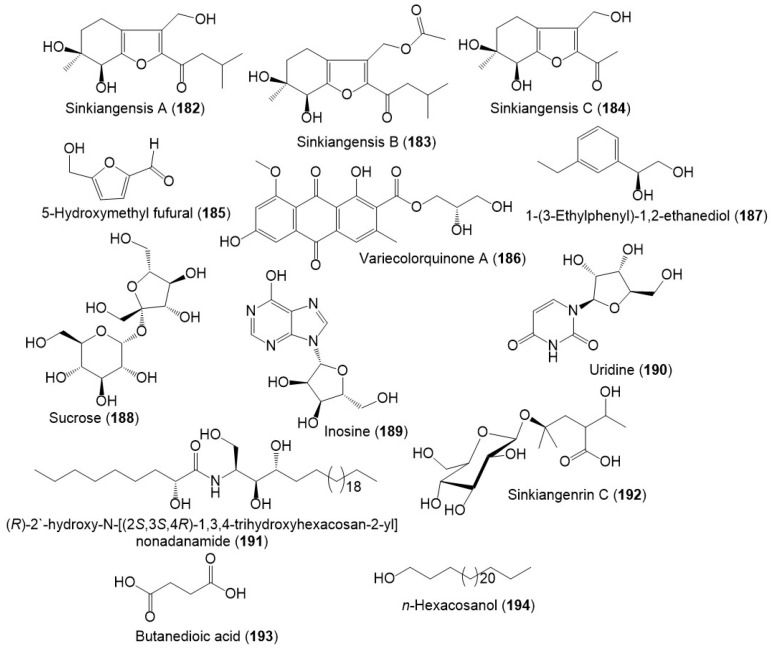
Structures of other metabolites (**182**–**194**) reported from *F. sinkiangensis*.

From the resin extract, new tetrahydrobenzofuran derivatives: sinkiangensis A–C (**182**–**184**) were purified using SiO_2_ CC/HPLC and elucidated by spectral and ECD analyses (Figure 20) [46]. Besides, sinkiangenrin C (**192**), a new organic acid glycoside was purified from seeds 95%EtOH extract. It is a 2-(1-hydroxyethyl)-4-methyl pentanoic acid 4-O-β-D-glucopyranoside [17].

### 5.10. Polysaccharides

Ghulameden et al., reported the separation of water-soluble polysaccharide from *F. sinkiangensis* roots gathered from Yili, Xinjiang Region, China using DEAE-cellulose-52 column (distilled H_2_O, as eluent) that was assigned by IR and HPLC analyses. The FSPs (crude polysaccharides) had ribose/arabinose/glucose/fucose/galactose (ratios 8.9/3.3/2.1/1.5/0.3), while FSPs-n (neutral polysaccharides) and FSPs-a (acidic polysaccharides) contained glucose/xylose/arabinose/galactose/mannose (ratios 3.9/4.0/1.8/1.4/0.8) and glucose/xylose/mannose/arabinose (ratios 6.5/4.0/1.7/1.0), respectively [34].

In another study, the sequential extraction of *F. sinkiangensis* roots yielded 28.86 wt% total polysaccharides. The polysaccharide fractions are heteropolysaccharides, containing galacturonic and glucuronic acids, galactose, xylose, rhamnose, fructose, and arabinose [47].

## 6. Biological Activities of *F. sinkiangensis* Extracts and Metabolites

### 6.1. Anti-Inflammatory and Anti-Neuroinflammatory Activity

From the Chinese medicine Awei (*F. sinkiangensis* gum resin) CHCl_3_ extract, new metabolites; **23** and **36**, in addition to formerly reported **1**, **5**, **7**, **8**, **11**–**13**, **16**, **32**, **33**, **39**, **41**, **52**, and **57** (Table 1)were assessed for their anti-neuroinflammatory activity against LPS (lipopolysaccharide)-stimulated NO production in BV-2 microglial cells using the nitrite and MTT (3-(4,5-dimethylthiazol-2-yl)- 2,5-diphenyltetrazoliumbromide) assays. It was noted that **7**, **12**, **32**, and **33** possessed marked activity (IC_50_s 6.93, 4.96, 10.5, and 5.95 µM, respectively) compared to minocycline (IC_50_ 37.04 µM), whereas **5**, **8**, **11**, **13**, **39**, **41**, and **57** greatly lessened NO production (IC_50_s ranged 19.88–47.43 µM). Compound **12** (Conc. 1–10 µM) remarkably suppressed IL-6, TNF-α, and IL-1β expression, as well as COX-2 (Conc. 3–10 µM) caused by LPS in BV2 cells. Thus, this plant might have the potential as anti-Alzheimer′s disease therapy [48]. Structure-activity relationship revealed that the substitution at C-3` in the bicyclic derivatives that possess 8`R-CH_3_ and 8`R-OH had a substantial role in the activity. The capability of C-3`-substitutents to boost the effect followed this order: acetoxy, α-OH, β-OH, and C=O, however, this order varied in bicyclic derivatives with C-8` terminal olefinic bond. In the mono-cyclic derivatives, the rings` breaking position of sesquiterpene moiety could influence the efficacy e.g., **32** and **33** with broken A-ring were more active than **39** and **41** with broken B-ring. Besides, the O-bridge in ring A in monocyclic derivatives improved the efficacy. On the other hand, the chain derivatives (e.g., **57** and **52**) had weak activity [48]. In 2020, Zhang et al., evaluated the potential of **12** on ischemic stroke utilizing BCCAO (bilateral common carotid artery occlusion) and LPS-invigorated microglia models. It was found that **12** relieved cognitive weakness, lowered neuronal forfeiture, repressed microglial stimulation, and converted microglia from the proinflammation M1 type to the anti-inflammation M2 type in the BCCAO-mice model. Moreover, it organized microglial polarization and suppressed the MAPK (mitogen-activated protein kinase) and NLRP3 signaling pathways subsequent to LPS-treatment in vitro. These findings highlighted the possible activity of **12** for treating ischemic stroke [49].

Further, in 2021, Mi et al., explored the potential of **12** on cerebral ischemia utilizing MCAO (middle-cerebral-artery occlusion) and LPS-boosted microglia models. In the MCAO model, **12** amended neurological outcomes and decreased infarct size and edema of the brain in rats. It also mitigated neuron injury and restrained microglial activation. Moreover, **12** guarded neuronal cells against damage by repressing microglial activation in LPS-invigorated BV2 cells. It also diminished the proinflammatory cytokines levels, NADPH oxidase activity, and ROS generation, along with the NF-κB signaling pathway repression [28].

Chemical investigation of *F. sinkiangensis* resin 95% EtOH extract resulted in new sesquiterpene coumarins: **27**, **28**, **31**, **38**, **46**, and **56**–**60,** along with early reported **3**, **5**, **8**, **23**, **25**, **29**, **30**, **40**, **41**, and **52**. They were characterized spectral/ECD/[α]_D_ analyses. Their inhibitory potential on NO production induced by LPS using nitrite and MTT assays in BV2 cells. Compounds **3**, **5**, **8**, **25**, **28**–**31**, **38**, **40**, **41**, **46**, and **56** possessed noticeable inhibition of NO production in over-activated BV2 cells, (IC_50_s 1.2–93.8 μM) compared to minocycline (IC_50_ 65.5 μM), while **27**, **23**, **52**, and **57–59** had weak inhibitory capacity (IC_50_ > 00 μM). It was noted that **28** (IC_50_ 1.2 μM) revealed the potent in vitro anti-neuroinflammatory that was confirmed by docking to TLR4/MD-2. Structure-activity relation showed that chain sesquiterpene coumarins with C-10` acetoxy group (e.g., **56**) had powerful activity than the ones with C-10`-OH, an unsaturated fatty chain or 4-decylenic acid ester (e.g., **57**, **58**, and **59**, respectively). A-ring substitution pattern affected the potential of monocyclic derivatives with opened B-ring. The α,β-unsaturated ketone (e.g., **38**) increased the effect, while the seven-membered ring-A resulted from C-4′-O–C–3` linkage (e.g., **28**) remarkably repressed NO producing relative to six-membered ring-A derivatives. In the compounds with opened A-ring (e.g., **29**–**31** and **46**), increase the length of oxyacyl side chain at C-3` weakened the anti-neuroinflammatory activity [25].

Among the lignan derivatives, **90**–**101**, **92** and **93** possessed anti-inflammatory potential via inhibiting TNF-α and IL-6 production mediated by LPS in RAW264.7 cells without affecting the RAW264.7 viability. Besides **92** and **93** notably suppressed LPS-produced iNOS and COX-2 expression in RAW264.7 cells [44], whereas **102**–**111** had COX-2 inhibition capacity (IC_50_s 4.47–21.96 μM) [18]. This evidence supported the role of *F. sinkiangensis* in treating inflammation [18,44].

In 2022, ferulagenol A (**176**) a new phenolic metabolite, in addition to **158**, **171**–**173**, **175**, and **177** were reported by Yan et al., Compounds **176** and **177** possessed notable COX-2 inhibitory capacity (IC_50_s 3.63 and 3.0 μM, respectively) [18].

On the other hand, *F. sinkiangensis* gum resin CHCl_3_ extract considerably prohibited production of NO in LPS-boosted BV-2 microglial cells (IC_50_ 1.66 µg/mL) [48].

### 6.2. Anticancer Activity

New sesquiterpene coumarins, **10**, **17**, **21**, and **22** and related analogs **1**, **5**–**9**, **11**–**14**, **20**, **24**, **26**, **29**, **30**, **32**, **37**, **39**, **41**, **52**, **54**, and **57** were reported from the 95% EtOH extract of the resin [39]. These compounds displayed moderate to weak anticancer potential against AGS, HeLa, and MGC-803 cell lines. Compounds **5**, **8**, and **29** demonstrated anticancer potential against AGS, HeLa, and MGC-803 cell lines (IC_50_s 20.0–49.0 μM), compared to taxol (IC_50_ 1.8, 7.5, and 3.4, respectively). Compound **10** had (IC_50_16.0 μM) selective activity against HeLa cells. The mechanistic study demonstrated that **10** caused G_0_/G_1_ cell cycle arrest and apoptosis in HeLa cells. It induced its effect by affecting the expression of cell cycle regulation- and apoptosis-related proteins by stimulating the MAPK pathway [39]. Additional work by Li et al., reported the separation of another new sesquiterpene coumarins, sinkiangenorin F (**42**) and its 8-acetyl derivative, and 8-O-acetyl sinkiangenorin F (**43**) from the seeds EtOH extract. They feature ether-linked coumarin and sesquiterpene moieties with 6`S/8`S/9`S. They exhibited anticancer potential (IC_50_s 27.1 and 62.7 µM, respectively) against AGS cell lines in the MTT assay [51].

**Scheme 4:**
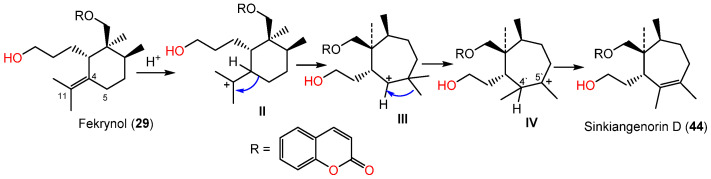
Biosynthetic pathway of sinkiangenorin D (**44**) [52].

Li et al. in 2015 purified and characterized sinkiangenorin D (**44**), a novel sesquiterpene coumarin having fekrynol- sesquiterpene skeleton, along with **2**, **15**, **19**, **24**, **25**, **29**, **34**, **35**, **41**, and **52** from the seeds’ EtOH extracts. Compound **44** is sesquiterpene coumarin involving a monocyclic cycloheptene sesquiterpene unit. These metabolites (IC_50_s 12.7–226.6 μM) demonstrated anticancer capacity on HeLa, AGS, and K562 in the MTT assay. Compounds **24**, **25**, **29**, **35**, **41**, and **44** had activity on HeLa cells (IC_50_s 20.4–226.2 μM), whereas **2**, **15**, **24**, **34**, **41**, **44**, and **52** were active against AGS cells (IC_50_s 12.7–104.8 μM) compared to taxol (IC_50_s 3.5–8.5 μM) [50]. Sinkiangenorin D (**44**) was proposed to be biosynthesized from fekrynol-kind sesquiterpene [50]. Firstly, the formation of **II** is accomplished by C-4 protonation and the olefinic bond electron-transport reaction. Thereafter, the C4–C5 electrons would transmit to C11, resulting in a seven-member ring formation. The C-5′ methyl transmission and successive loss of proton form C4′–C5′ double bond result in this novel framework formation [50] (Scheme 4).

A novel metabolite (**45**) having unrivaled bicyclo[4.3.1]decane-type sesquiterpene skeleton was purified from the 95% EtOH extract of the seeds. Its 2′S/3′R/5′R/7′R/10′R configuration was determined based on NMR and CD analyses. It had moderate and weak anticancer activity against AGS (IC_50_ 12.7 μM) and Hela (IC_50_ 82.9 μM) cells, respectively compared to taxol (IC_50_ 3.5 and 5.6 μM, respectively) [52].

Umbelliprenin (**52**) possessed dose-dependent and time-dependent apoptosis in CLL (chronic lymphocytic leukemia) that was more sensitive to **52** than PBMCs. It is noteworthy that IL-4 could not decline **52**-caused apoptosis in CLL (Figure 7). Thus, **52** oral administration as foods or folk medicines, might stimulate the protection against CLL development with few side effects, however, additional clinical investigations are needed [53]. Gholami et al., reported that **52** potentiated apoptosis intrinsic/extrinsic pathways in Jurkat cells by activating caspase-9 and -8, as well as Bcl-2 prohibition [54]. Another study by Zhang et al., revealed that **52** had a notable anticancer activity (IC_50_s 13.67 and 20.82 µM, respectively) against AGS cells with less anticancer to GES-1 (normal human gastric epithelial cell line). It boosted AGS cells apoptosis with elevated Bax/Bcl-2 ratios, ROS generation, lessened mitochondrial-membrane potential, and PARP and caspase 3 activation resulting in mitochondrial apoptosis pathway activation. It also arrested the G0/G1 phase of the cell cycle, increased P27, P53, and P16, expression, and diminished cyclin E, cyclin D, Cdk2, and Cdk4 expression in cancer cells. Therefore, it could be developed into anti-cancer therapy [24]. In 2019, Zhang et al., also reported that **52** also demonstrated anticancer capacity against BGC-823 and AGS, with less toxic influence on the normal GES-1 gastric cells. It was proven to prevent gastric cancer cell invasion, growth, and migration by disconcerting the Wnt signaling pathway. Additionally, it exhibited no harm in the in vivo BGC-823 xenograft model as evidenced by no observed abnormality in daily diet, body weight, liver function, and histological features of the spleen, liver, lung, kidney, and heart tissue. This further supported the previous evidence of its promising potential for treating gastric cancer [55].

The anticancer investigation against SW1990, CFPAC-1, Capan-2, and PANC-1 revealed that (+)-**61** and (-)-**62** had marked proliferation inhibition capacity on PANC-1 cells (IC_50_s 2.24 and 0.92 μM, respectively) and moderate activity against CFPAC-1 cells (IC_50_s 6.12 and 19.13 μM, respectively) compared to taxol (IC_50_s 0.22 and 0.38 μM, respectively). It was noted that the anticancer potential of (+)-**61** was more powerful than (-)-**62** [40]. Also, **63**–**65** had anticancer activity (EC_50_s 22.78–65.38 μM) against IOZCAS-Spex-II, where **65** was the most potent (EC_50_ 22.78 μM) relative to camptothecin (EC_50_ 51.27 μM) [41].

Wang et al., examined the activity of **69** and **70** against AGS, MGC-803, and GES-1 using MTT assay. Only **70** possessed the notable anticancer potential against MGC-803 and AGS (IC_50_s 18.89 and 16.15, respectively) with less toxicity against GES-1 (IC_50_ 36.73 μM) comparing with taxol (IC_50_ 3.35, 1.82, and 2.67 μM, respectively), whereas the other metabolites had no notable potential. The mechanistic study revealed that **70** was found to elevate Bax/Bcl-2 ratios, as well as RB, P16, and P27 expression and decrease cyclins (D1 and E), Cdk4, P53, and Cdk2, resulting in apoptosis and G_0_/G_1_ cell cycle arrest in AGS cells. This compound could be a potential candidate for gastric cancer therapy [29].

*F. sinkiangensis* seeds afforded **77**–**80** that displayed weak to moderate anticancer activity on AGS and Hela cells in the MTT assay [17] (Figure 10), whereas **77**, **80**, and **81** had a weak anticancer capacity against AGS, HeLa, A549, and PC3 cell lines in the MTT assay (IC_50_s 54.4–167.3 µM) [24].

In the CCK-8 assay, **84** and **86** were found to significantly prohibit the migration and invasion of TNBC) cell lines. On the other hand, **88** and **89** promoted the HUVECs) proliferation which was more remarkable than bFGF (basic-fibroblast-growth factor, positive control) in the wound-healing assay [35].

New phenylpropanoid derivative; sinkiangenones C (**179**) and D (**180**), along with **158**, **163, 164**, **166**–**170, 174**, and **181** were separated from the resin 95% EtOH extract and specified by spectral and CD analyses. In the MTT assay against AGS, MGC-803, and GES-1, they had no or weak potential (IC_50_s 18.89 to 182.46 μM) [29].

Sinkiangensis A**–**C (**182–184**) possessed anticancer activity on the AGS cell line (IC_50_s 87.1, 72.7, and 15.6 μM, respectively), whereas **184** was the most active in comparison to taxol (IC_50_ 4.69 μM) and induced AGS cell apoptosis in the MTT assay. Unfortunately, they (IC_50_ ˃100 μM) had no effect against HeLa and K562 cells [46]. Whilst **192** exhibited anticancer activity on AGS cells (IC_50_ 36.9 μM) [17].

The petroleum ether, EtOAc, n-BuOH, and MeOH fraction possessed of *F. sinkiangensis* resin anticancer activity against Caco-2, HC-T116, MFC, and HepG2 cells in the SRB assay. EtOAc fraction was found to have the potent anti-proliferative and apoptotic effects against all tested cell lines. This was correlated to its content of sesquiterpene coumarins [31].

### 6.3. Antiviral, Insecticidal, and Antimicrobial Activities

Besides, **45** prohibited (IC_50_ 4.0 μM) BJ09/H1N1 (influenza A/Beijing/7/2009 H1N1) infection in MDCK cells [52]. The monoterpene coumarins; **63**–**65** and the coumarins: **66** and **67** displayed insecticidal potential (Conc. 10 μg/larva, 24 h) against *S. exigua* 3rd instar larvae (%mortality ranging from 26.67–52.22%) compared to camptothecin (18.89% mortality) [41]. From the aerial parts, sesquiterpenes; nerolidol (**120**) and guaiol (**122**) (58.89 and 41.11% mortality, respectively) possessed toxic potential on the *S. exigua* insect 3rd instar larvae [41]. Additionally, **120** exhibited antifungal effect against plant pathogens: inhibitory effects on *Pyricularia grisea, Alternaria alternata,* and *Botrytis cinereal* (MICs 16, 32, and 32 μg/mL, respectively) compared to carbendazim (MICs 32, 16, and 32 μg/mL, respectively) [41].

### 6.4. Anti-Drug Addiction Activity

Drug addiction is a prime health concern that influences a growing number of persons and gives rise to severe economic and social burdens to economy society [56,57]. Despite the fact that diverse remedial strategies for drug addiction and abuse are developed, including psychological, sociological, and pharmacological interventions, their activity is yet restricted [58,59].

A mixture of **133** and **135** was obtained from the *F. sinkiangensis* crude essential oil. A mixture of **133** and **135** (1:3 ratio, doses: 20, 40, and 60 mg/kg, ip) significantly repressed the morphine abstinence syndrome and physiological addiction in rats and mice [60]. At the same doses, this mixture (1:3 ratio, i.p.) reduced morphine-induced bodyweight loss. While the mixture declined the abdominal writhing movements number and automatic activity (doses 10.73, 21.45, and 43.55 mg/kg, i.p.) revealing its analgesic and sedative potential. Its acute toxicity evaluation showed the LD_50_ values of its iv and ip injections were 1.42 g/kg and 1.66 g/kg, respectively [60].

In 2006, Wang reported in his patent that the resin extract in the form of capsules, powder, injection, drop pills, granules, tablets, or oral liquid) ameliorated the influences of serious and moderate long-time drug addictions in addicted patients, indicating its potential for treating subjects addicted to morphine, opioid, diamorphine, and marijuana [36,38]. It is noteworthy that 0.1–20 g/kg was found to be the therapeutically effective amount of the extract for producing an effect of abstinence of morphine, whereas the preferable dose was 1–3 g/kg [36].

### 6.5. *Protein-Tyrosine Phosphatase 1B Inhibition Activity*

FSPs-a (acidic polysaccharides) fraction of *F. sinkiangensis* roots water-soluble polysaccharides revealed in vitro PTP1B (protein-tyrosine phosphatase 1B) competitive inhibition (IC_50_ 0.29 µg/mL, % inhibition 91.23%) [34]. In another study, the PTP1B inhibitory potential of the different polysaccharides fractions was estimated. It was noted that the inhibitory capacity of the tested fractions elevated with raising their galactose content, therefore, galactose may be a ligand for blocking PTP1B catalytic site [47].

### 6.6. Antiulcer and Antioxidant Activities

In the in vivo antiulcer assay, different *F. sinkiangensis* resin extracts possessed antiulcer capacity, whereas CHCl_3_ extract (%inhibition 48.43) had comparatively better antiulcer potential than the *n*-BuOH and EtOAC (%inhibition 37.07 and 46.06%, respectively) extracts comparing to famotidine (%inhibition 45.37) [14]. In the DPPH assay, the *F. sinkiangensis* resin *n*-BuOH, EtOAc, and MeOH fractions significantly scavenged DPPH, whereas the EtOAc fraction was the most effective and the petroleum ether fraction was weakly active in the DPPH assay [31].

### 6.7. Feed Attraction Activity

In 2020, Xu et al., reported that feeding *Lateolabrax japonicus* (commercial fish) with *F. sinkiangensis* was found to notably promote *L. japonicus* foraging and better digestive enzyme activity and fish growth performance, where 10 g/kg was appropriate in the fish diet. Thus, it had an efficient role in *L. japonicus* farming and could have potential in the aquaculture industry as aquafeed formulation [61].

## 7. Traditional Ethnomedicinal Uses in Asian Countries

Medicinal plants are fundamental to humans and utilized for thousands of years in various cultures to treat or prevent diseases or promote health and well-being [62]. Many communities continue to depend on plants as the main tool for healing various illnesses and have established their medical systems on the basis of their unique beliefs, experiences, and theories worldwide [63]. Traditional and indigenous medical systems are especially widespread throughout communities in Asia that are accountable for a remarkable proportion of the healthcare provided in these countries [64,65]. Ayurveda, Jamu, traditional Philippines, traditional Malay, Sowa Rigpa, Tibetan, Kampo, Siddha, Thai medicine, Unani, and traditional Chinese systems are important sources of livelihood and health for millions of Asian people [62,66]. Generally, the region’s traditional medicine systems are greatly affected by those practiced in the neighboring areas especially of South and East Asia, mainly that of India and China [62]. In China, different sociolinguistic groups have their own traditional systems and medicinal plant usages that vary based on associated ecology and geography [67]. For example, Southwest China (kingdom of plants) is renowned for its large variety of ethnic groups with featured traditional cultures. Populations from 33 ethnicities are using plants as a traditional remedy for thousands of years, including Bai, Achang, Bulang, Tibetan, Buyi, Dai, Dong, De’ang, Hani, Dulong, Hui, Han, Jinuo, Lahu, Jingpo, Lisu, Maonan, Luoba, Menba, Molao, Miao, Naxi, Pumi, Nu, Qiang, Shui, She, Tujia, Yao, Wa, Yi, Zhuang, and Gelao people [67,68].

## 8. Conclusions

**Figure 21:**
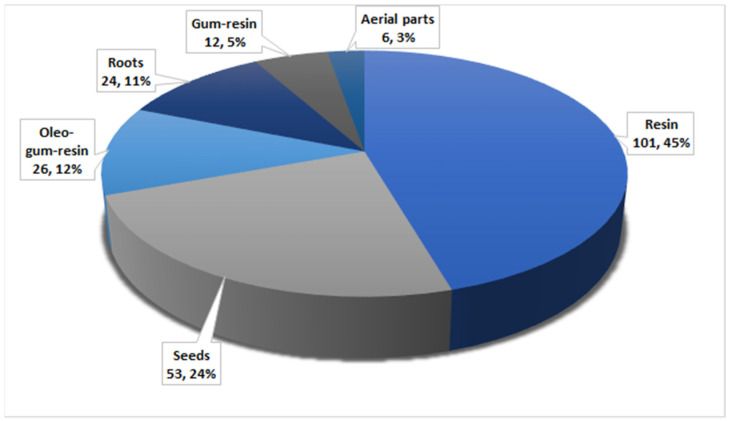
Number of compounds and their percentage reported from various parts of *F. sinkiangensis*.

Herbal medicines have been utilized for thousands of years as principal therapeutic agents for treating various human illnesses in many countries. Recently, a growing number of studies have been carried out to prove the efficacy of these medicines against the assigned disorders. *F. sinkiangensis* is among the most valuable species of the genus *Ferula* that possess various traditional applications in treating various disorders such as bronchitis, diarrhea, malaria, gastric disorders, and rheumatoid arthritis. In this work, 194 metabolites have been characterized from various parts of this plant, including aerial parts, seed, roots, gum resin, oleo-gum resin, and resins (Figure 21).

**Figure 22:**
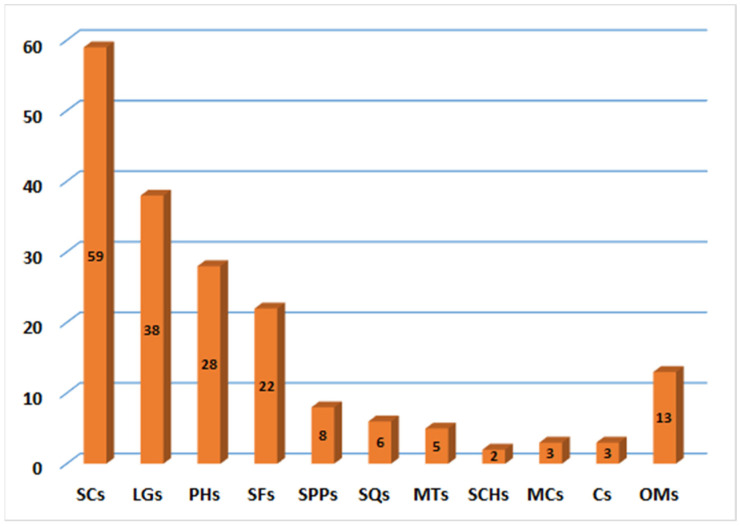
Number of compounds reported from various classes of *F. sinkiangensis*. SCs: Sesquiterpene coumarins; LGs: Lignans; PHs: Phenolics; SFs: Sulfanes; SPPs: Sesquiterpene phenylpropanoids; SQs: Sesquiterpenes; MTs: monoterpenes; SCHs: Sesquiterpene chromones; MCs: Monoterpene coumarins; Cs: coumarins; OMs: Other metabolites.

It was obvious that the majority of metabolites have been distinguished from resin extract. These metabolites belong to various chemical classes. Sesquiterpene coumarins with their structural diversity and contents represent the main and substantial metabolites of this plant (Figure 22).

Many studies surmised that these compounds may have a substantial contribution in many of the reported activities of *F. sinkiangensis*. *F. sinkiangensis* had metabolites with marked antifungal and insecticidal capacity that can be valuable in agriculture for insect and plant pathogens control, however, additional field assessment is requested. Its metabolites; **61**, **62**, **65**, **70**, and **120** had notable anticancer potential against different cancer cell lines. These findings would provide evidence for the application of *this* and its fractions in treating cancers. Compound **12** had marked anti-inflammatory and anti-neuroinflammatory potential, revealing its potential as a lead metabolite for therapeutic intervention in various illnesses such as ischemic stroke, Alzheimer`s disease, and cerebral ischemia. Many studies proved the anticancer and apoptotic potential of **52** against different cancer cell lines particularly the gastric cancer cells with no toxic effect on the normal cells and no observed abnormality in daily diet, body weight, liver function, and histological features of the spleen, liver, lung, kidney, and heart tissue. This further supported its promising potential for treating gastric cancer as foods or folk medicine, however, additional clinical investigations are needed. To find out more metabolites with unique structures and bioactivity, more phytochemical investigations are demanded and indispensable. Also, new technologies such as metabolomics, transcriptomics, genomics, and proteomics can be applied for discovering more metabolites from this valuable medicinal plant. The plant`s mechanism in treating gastric disorders and rheumatoid arthritis is insufficiently explored. Additionally, in-depth in-vivo and in vitro studies of the other bio-activities mechanisms are required. Meanwhile, toxicological, pharmacokinetic, preclinical, quality control, and clinical studies are insistent to estimate the safety and rationale usage of this plant. Finally, the integration of traditional knowledge into ecology-based research for the endangered medicinal plant protection must be promoted.

## Figures and Tables

**Table 1:** Biological activity of reported metabolites from Ferula sinkiangensis.

Compound Name	Biological Activity	Assay, Organism or Cell Line	Biological Results		Ref.
			Compound	Positive Control	
Farnesiferol A (**1**)	Anticancer	MTT/HeLa	20.0 µM (IC_50_)	Taxol 7.5 µM (IC_50_)	[39]
Colladonin (**2**)	Anticancer	MTT/AGS	85.5 µM (IC_50_)	Taxol 3*.5* µM (IC_50_)	[50]
Coladin (**3**)	Anti-neuroinflammatory	Griess reaction/NO production/LPS-activated BV-2 cells	60.5 µM (IC_50_)	Minocycline 65.5 µM (IC_50_)	[25]
Farnesiferone A (**5**)	Anti-neuroinflammatory	Griess reaction/NO production/LPS-activated BV-2 cells	37.88 µM (IC_50_)	Minocycline 37.04 µM (IC_50_)	[48]
		Griess reaction/NO production/LPS-activated BV-2 cells	37.9 µM (IC_50_)	Minocycline 65.5 µM (IC_50_)	[25]
	Anticancer	MTT/HeLa	23.0 µM (IC_50_)	Taxol 7.5 µM (IC_50_)	[39]
		MTT/MGC-803	49.0 µM (IC_50_)	Taxol 3.4 µM (IC_50_)	[39]
		MTT/AGS	32.0 µM (IC_50_)	Taxol 1.8 µM (IC_50_)	[39]
Gummosin (**7**)	Anti-neuroinflammatory	Griess reaction/NO production/LPS-activated BV-2 cells	6.93 µM (IC_50_)	Minocycline 37.04 µM (IC_50_)	[48]
Polyanthinin (**8**)	Anti-neuroinflammatory	Griess reaction/NO production/LPS-activated BV-2 cells	19.88 µM (IC_50_)	Minocycline 37.04 µM (IC_50_)	[48]
		Griess reaction/NO production/LPS-activated BV-2 cells	37.3 µM (IC_50_)	Minocycline 65.5 µM (IC_50_)	[25]
	Anticancer	MTT/HeLa	28.0 µM (IC_50_)	Taxol 7.5 µM (IC_50_)	[39]
	Anticancer	MTT/MGC-803	45.0 µM (IC_50_)	Taxol 3.4 µM (IC_50_)	[39]
	Anticancer	MTT/AGS	24.0 µM (IC_50_)	Taxol 1.8 µM (IC_50_)	[39]
Sinkiangenol E (**10**)	Anticancer	MTT/HeLa	16.0 µM (IC_50_)	Taxol 7.5 µM (IC_50_)	[39]
Ferukrin (**11**)	Anti-neuroinflammatory	Griess reaction/NO production/LPS-activated BV-2 cells	21.34 µM (IC_50_)	Minocycline 37.04 µM (IC_50_)	[48]
(3`*S*, 5`*S*, 8`*R*, 9`*S*, 10`*R*)-Kellerin (**12**)	Anti-neuroinflammatory	Griess reaction/NO production/LPS-activated BV-2 cells	4.96 µM (IC_50_)	Minocycline 37.04 µM (IC_50_)	[48]
	Anticancer	MTT/HeLa	37.0 µM (IC_50_)	Taxol 7.5 µM (IC_50_)	[39]
(3`*S*, 5`*S*, 8`*R*, 9`*S*, 10`*R*)-Deacetylkellerin (**13**)	Anti-neuroinflammatory	Griess reaction/NO production/LPS-activated BV-2 cells	31.61 µM (IC_50_)	Minocycline 37.04 µM (IC_50_)	[48]
Episamarcandin (**15**)	Anticancer	MTT/AGS	83.8 µM (IC_50_)	Taxol 3*.5* µM (IC_50_)	[50]
Lehmannolol (**24**)	Anticancer	MTT/AGS	26.0 µM (IC_50_)	Taxol 3*.5* µM (IC_50_)	[50]
	Anticancer	MTT/HeLa	42.0 µM (IC_50_)	Taxol 7.5 µM (IC_50_)	[39]
Lehmannolone (**25**)	Anticancer	MTT/HeLa	81.1 µM (IC_50_)	Taxol 5.6 µM (IC_50_)	[50]
	Anti-neuroinflammatory	Griess reaction/NO production/LPS-activated BV-2 cells	93.8 µM (IC_50_)	Minocycline 65.5 µM (IC_50_)	[25]
Ferusingensine H (**28**)	Anti-neuroinflammatory	Griess reaction/NO production/LPS-activated BV-2 cells	18.6 µM (IC_50_)	Minocycline 65.5 µM (IC_50_)	[25]
Fekrynol (**29**)	Anti-neuroinflammatory	Griess reaction/NO production/LPS-activated BV-2 cells	13.0 µM (IC_50_)	Minocycline 65.5 µM (IC_50_)	[25]
	Anticancer	MTT/HeLa	35.0 µM (IC_50_)	Taxol 7.5 µM (IC_50_)	[39]
		MTT/MGC-803	49.0 µM (IC_50_)	Taxol 3.4 µM (IC_50_)	[39]
		MTT/AGS	20.0 µM (IC_50_)	Taxol 1.8 µM (IC_50_)	[39]
Fekrynol acetate (**30**)	Anti-neuroinflammatory	Griess reaction/NO production/LPS-activated BV-2 cells	15.7 µM (IC_50_)	Minocycline 65.5 µM (IC_50_)	[25]
	Anticancer	MTT/HeLa	25.0 µM (IC_50_)	Taxol 7.5 µM (IC_50_)	[39]
		MTT/MGC-803	28.0 µM (IC_50_)	Taxol 3.4 µM (IC_50_)	[39]
(8`S, 9`S, 10`S)-Propionyl fekrynol (**31**)	Anti-neuroinflammatory	Griess reaction/NO production/LPS-activated BV-2 cells	21.3 µM (IC_50_)	Minocycline 65.5 µM (IC_50_)	[25]
Galbanic acid (**32**)	Anti-neuroinflammatory	Griess reaction/NO production/LPS-activated BV-2 cells	10.50 µM (IC_50_)	Minocycline 37.04 µM (IC_50_)	[48]
	Anticancer	MTT/HeLa	43.0 µM (IC_50_)	Taxol 7.5 µM (IC_50_)	[39]
Methyl galbanate (**33**)	Anti-neuroinflammatory	Griess reaction/NO production/LPS-activated BV-2 cells	5.95 µM (IC_50_)	Minocycline 37.04 µM (IC_50_)	[48]
Fekolone (**34**)	Anticancer	MTT/AGS	75.4 µM (IC_50_)	Taxol 3*.5* µM (IC_50_)	[50]
Sinkianone (**35**)	Anticancer	MTT/HeLa	77.9 µM (IC_50_)	Taxol 5.6 µM (IC_50_)	[50]
Ferusingensine G (**38**)	Anti-neuroinflammatory	Griess reaction/NO production/LPS-activated BV-2 cells	1.2 µM (IC_50_)	Minocycline 65.5 µM (IC_50_)	[25]
Farnesiferol B (**39**)	Anti-neuroinflammatory	Griess reaction/NO production/LPS-activated BV-2 cells	45.37 µM (IC_50_)	Minocycline 37.04 µM (IC_50_)	[48]
Farnesiferone B (**40**)	Anti-neuroinflammatory	Griess reaction/NO production/LPS-activated BV-2 cells	18.3 µM (IC_50_)	Minocycline 65.5 µM (IC_50_)	[25]
Farnesiferol C (**41**)	Anti-neuroinflammatory	Griess reaction/NO production/LPS-activated BV-2 cells	31.43 µM (IC_50_)	Minocycline 37.04 µM (IC_50_)	[48]
		Griess reaction/NO production/LPS-activated BV-2 cells	22.6 µM (IC_50_)	Minocycline 65.5 µM (IC_50_)	[25]
	Anticancer	MTT/HeLa	86.9 µM (IC_50_)	Taxol 5.6 µM (IC_50_)	[50]
	Anticancer	MTT/AGS	101.6 µM (IC_50_)	Taxol 3*.5* µM (IC_50_)	[50]
	Anticancer	MTT/HeLa	25.0 µM (IC_50_)	Taxol 7.5 µM (IC_50_)	[39]
Sinkiangenorin F (**42**)	Anticancer	MTT/AGS	27.1 µM (IC_50_)	Taxol 3*.5* µM (IC_50_)	[51]
8-O-Acetyl sinkiangenorin F (**43**)	Anticancer	MTT/AGS	62.78 µM (IC_50_)	Taxol 3*.5* µM (IC_50_)	[51]
Sinkiangenorin D (**44**)	Anticancer	MTT/HeLa	20.4 µM (IC_50_)	Taxol 5.6 µM (IC_50_)	[50]
	Anticancer	MTT/K562	81.1 µM (IC_50_)	Taxol 8.5 µM (IC_50_)	[50]
	Anticancer	MTT/AGS	104.8 µM (IC_50_)	Taxol 3*.5* µM (IC_50_)	[50]
Sinkiangenorin E (**45**)	Anticancer	MTT/AGS	12.7 µM (IC_50_)	Taxol 3*.5* µM (IC_50_)	[52]
		MTT/HeLa	82.9 µM (IC_50_)	Taxol 5.6 µM (IC_50_)	[52]
	Antiviral	A/Beijing/7/2009 H1N1 (BJ09/H1N1)	4.0 µM (IC_50_)	*-*	[52]
Ferusingensine F (**46**)	Anti-neuroinflammatory	Griess reaction/NO production/LPS-activated BV-2 cells	29.0 µM (IC_50_)	Minocycline 65.5 µM (IC_50_)	[25]
Umbelliprenin (**52**)	Anticancer	MTT/AGS	12.7 µM (IC_50_)	Taxol 3*.5* µM (IC_50_)	[50]
10*R*-Karatavicinol (**53**)	Anti-neuroinflammatory	Griess reaction/NO production/LPS-activated BV-2 cells	47.43 µM (IC_50_)	Minocycline 37.04 µM (IC_50_)	[48]
12′-Hydroxy-karatavicinol (**54**)	Anticancer	MTT/HeLa	48.0 µM (IC_50_)	Taxol 7.5 µM (IC_50_)	[39]
Ferusingensine A (**56**)	Anti-neuroinflammatory	Griess reaction/NO production/LPS-activated BV-2 cells	31.8 µM (IC_50_)	Minocycline 65.5 µM (IC_50_)	[25]
Ferusingensine E (**60**)	Anti-neuroinflammatory	Griess reaction/NO production/LPS-activated BV-2 cells	65.4 µM (IC_50_)	Minocycline 65.5 µM (IC_50_)	[25]
(+)-Ferulasin 2*R*, 3*R*, 10`*R* (**61**)	Anticancer	MTT/SW1990	11.77 µM (IC_50_)	Taxol 1.70 µM (IC_50_)	[40]
	Anticancer	MTT/PANC-1	2.24 µM (IC_50_)	Taxol 0.22 µM (IC_50_)	[40]
	Anticancer	MTT/CFPAC-1	6.12 µM (IC_50_)	Taxol 0.38 µM (IC_50_)	[40]
	Anticancer	MTT/Capan-2	8.57 µM (IC_50_)	Taxol 0.51 µM (IC_50_)	[40]
(-)-Ferulasin 2*S*, 3*S*, 10`*S* (**62**)	Anticancer	MTT/PANC-1	0.92 µM (IC_50_)	Taxol 0.22 µM (IC_50_)	[40]
	Anticancer	MTT/CFPAC-1	19.13 µM (IC_50_)	Taxol 0.38 µM (IC_50_)	[40]
Ferusinkin A (**63**)	Anticancer	Spectrophotometrically/IOZCAS-Spex-II	65.38 µM (EC_50_)	Camptothecin 51.27 µM (EC_50_)	[41]
Diversin (**64**)	Anticancer	Spectrophotometrically/IOZCAS-Spex-II	52.67 µM (EC_50_)	Camptothecin 51.27 µM (EC_50_)	[41]
Auraptene (**65**)	Anticancer	Spectrophotometrically/IOZCAS-Spex-II	22.78 µM (EC_50_)	Camptothecin 51.27 µM (EC_50_)	[41]
Sinkiangenone A (**69**)	Anticancer	MTT/MGC-803	45.05 µM (IC_50_)	Taxol 3.35 µM (IC_50_)	[29]
	Anticancer	MTT/AGS	48.13 µM (IC_50_)	Taxol 1.82 µM (IC_50_)	[29]
	Anticancer	MTT/GES-1	32.37 µM (IC_50_)	Taxol 2.67 µM (IC_50_)	[29]
Sinkiangenone B (**70**)	Anticancer	MTT/MGC-803	18.89 µM (IC_50_)	Taxol 3.35 µM (IC_50_)	[29]
	Anticancer	MTT/AGS	16.15 µM (IC_50_)	Taxol 1.82 µM (IC_50_)	[29]
	Anticancer	MTT/GES-1	36.73 µM (IC_50_)	Taxol 2.67 µM (IC_50_)	[29]
Ferulaeone G (**72**)	Anticancer	MTT/MGC-803	35.15 µM (IC_50_)	Taxol 3.35 µM (IC_50_)	[29]
	Anticancer	MTT/GES-1	35.23 µM (IC_50_)	Taxol 2.67 µM (IC_50_)	[29]
Arctigenin (**77**)	Anticancer	MTT/AGS	78.3 µM (IC_50_)	Taxol 3.5 µM (IC_50_)	[17]
	Anticancer	MTT/HeLa	105.1 µM (IC_50_)	Taxol 5.6 µM (IC_50_)	[17]
Matairesinol (**78**)	Anticancer	MTT/AGS	99.4 µM (IC_50_)	Taxol 3.5 µM (IC_50_)	[17]
Nerolidol (**120**)	Anticancer	Spectrophotometrically/IOZCAS-Spex-II	14.64 µM (EC_50_)	Camptothecin 51.27 µM (EC_50_)	[41]
	Antifungal	Microdilution/*Alternaria alternata*	32.0 μg/mL (MIC)	Carbendazim 16.0 μg/mL (MIC)	[41]
		Microdilution/*Pyricularia grisea*	16.0 μg/mL (MIC)	Carbendazim 32.0 μg/mL (MIC)	[41]
		Microdilution/*Botrytis cinerea*	32.0 μg/mL (MIC)	Carbendazim 32.0 μg/mL (MIC)	[41]
Guaiol (**122**)	Anticancer	Spectrophotometrically/IOZCAS-Spex-II	88.92 µM (EC_50_)	Camptothecin 51.27 *µ*M (EC_50_)	[41]
Coniferaldehyde (**167**)	Anticancer	MTT/MGC-803	69.65 µM (IC_50_)	Taxol 3.35 µM (IC_50_)	[29]
Sinkiangensis A (**182**)	Anticancer	MTT/AGS	87.1 µM (IC_50_)	Taxol 4.69 µM (IC_50_)	[46]
Sinkiangensis B (**183**)	Anticancer	MTT/AGS	72.7 µM (IC_50_)	Taxol 4.69 µM (IC_50_)	[46]
Sinkiangensis C (**184**)	Anticancer	MTT/AGS	15.6 µM (IC_50_)	Taxol 4.69 µM (IC_50_)	[46]
Sinkiangenrin C (**192**)	Anticancerity	MTT/AGS	36.9 *µ*M (IC_50_)	Taxol 3.5 µM (IC_50_)	[17]

## Data Availability

Not applicable.

## Supplementary Materials

The following supporting information can be downloaded at: https://www.mdpi.com/article/10.3390/plants12040902/s1, Table S1: Secondary metabolites reported from Ferula sinkiangensis (Chemical class, molecular weight and formulae, plant part, and origin). References [8,13,14,17,18,19,24,25,27,29,30,35,39,40,41,43,44,46,48,50,51,52,60] are cited in the Supplementary Materials.

## Abbreviations

1D NMR	One-dimensional nuclear magnetic resonance
2D NMR	Two-dimensional nuclear magnetic resonance
A549	Human lung adenocarcinoma epithelial cell line
AGS	Human gastric carcinoma cell line
Bax/Bcl-2	B-cell lymphoma protein 2 (Bcl-2)-associated X (Bax)
BCCAO	Bilateral common carotid artery occlusion
bFGF	Basic fibroblast growth factor
BGC-823	Human gastric carcinoma cell line
n-BuOH	n-Butanol
BV-2	Microglia cells
Caco-2	Human colon adenocarcinoma cell line
Capan-2	Human pancreatic cancer cell line
CCK-8	Cell counting kit-8
CD	Circular dichroism
Cdk2	Cyclin dependent kinase 2
CFPAC-1	Human pancreatic cancer cell line
CHCl_3_	Chloroform
CLL	Chronic lymphocytic leukemia
COX-2	Cyclooxygenase-2
CITES	Convention on International Trade in Endangered Species of Wild Fauna and Flora
DCFH-DA	2′, 7′-Dichlorofluorescein diacetate
DEAE-Cellulose 52	Diethylaminoethyl cellulose-52
DPPH	1,1-Diphenyl-2-picrylhydrazyl
EC_50_	Half maximal effective concentration
ECD	Electronic circular dichroism
ELISA	Enzyme-linked immunosorbent assay
EtOH	Ethanol
EtOAc	Ethyl acetate
HR-ESIMS	High resolution electrospray ionization mass spectrometry
GES-1	Human normal gastric epithelial cell line
GSK-3β	Glycogen synthase kinase-3β
H_2_O	Water
HCT-116	Human colon cancer cell line
HepG2	Human hepatocellular liver carcinoma cell line
HeLa	Human cervical epitheloid carcinoma cell line
HPLC	High-performance liquid chromatography
HUVECs	Human umbilical vein endothelial cell line
IC_50_	Half-maximal inhibitory concentration
LD_50_	Lethal dose 50,
IL-6	Interleukin-6
IL-1β	Interleukin-1beta
IR	Infrared
IOZCAS-Spex-II	A cell strain cloned from Spodoptera exigua cell line
IUCN	International Union for Conservation of Nature
K562	Human erythroleukemic cell line
IR	Infrared
iNOS	Inducible nitric oxide synthase
LPS	Lipopolysaccharide
MAPK	Mitogen-activated protein kinase
MCAO	Middle cerebral artery occlusion
MDCK	Madin-Darby Canine Kidney
MeOH	Methanol
MFC	Mouse forestomach cancer cell line
MIC	Minimum inhibitory concentrations
MGC-803	Human gastric cancer cell line
MMP2 and MMP9	matrix metalloproteinases
MS	Mass spectrometry
MTT	3-(4,5-Dimethylthiazol-2-yl)-2,5-diphenyltetrazolium bromide
NADPH	Nicotinamide adenine dinucleotide phosphate
NBT	Nitrotetrazolium blue chloride
NLRP3	NLR family pyrin domain containing 3
NO	Nitric oxide
P16	Protein regulating the cell circle
P27	A key protein that regulator of cell proliferation
P53	Tumor suppressor protein
PANC-1	Human pancreas ductal carcinoma cell line
PARP	Poly (ADP-ribose) polymerase
PC-3	Human prostatic-testosterone-independent cell line
PCR	Polymerase chain reaction
PTP1B	Protein tyrosine phosphatase 1B
qRT-PCR	Quantitative real-time polymerase chain reaction
RAW264.7	Mouse macrophage cell line
ROS	Reactive oxygen species
RP-18	Reversed-phase-18
SCs	Sesquiterpene coumarins
SRB	Sulforhodamine B
SiO_2_ CC	Silica gel column chromatography
SW1990	Human pancreatic cancer cell line
TCF/LEF	T-cell factor/lymphoid enhancer factor
TLC	Thin layer chromatography
TLR4	Toll-like receptor 4
TNBC	Triple-negative breast cancer
TNF-α	Tumor necrosis factor alpha
TTC	2, 3, 5-Triphenyltetrazoliumchloride
UV	Ultraviolet
Wnt	Wingless-related integration site
