# Supplementary material for: Ferula sinkiangensis (Chou-AWei, Chinese Ferula): Traditional Uses, Phytoconstituents, Biosynthesis, and Pharmacological Activities
Source: Plants (Basel). 2023 Feb 16;12(4):902. doi: 10.3390/plants12040902 (PMC9958602; doi:10.3390/plants12040902)
Supplement: Supplementary file 1 [file plants-12-00902-s001.zip › plants-2143860-supplementary.pdf]

plants

MDPI

Supplementary Materials

Review

Ferula sinkiangensis (Chou-AWei, Chinese *Ferula*): Traditional Uses, Phyto-constituents, Biosynthesis, and Pharmacological Activities

Maan T. Khayat [1], Majed Alharbi,[1] Kholoud F. Ghazawi [2], Gamal A. Mohamed [3,] and Sabrin R. M. Ibrahim[4,5,*]

[1] Department of Pharmaceutical Chemistry, Faculty of Pharmacy, King Abdulaziz University, Jeddah 21589, Saudi Arabia

[2] Clinical Pharmacy Department, College of Pharmacy, Umm Al-Qura University, Makkah 24382, Saudi Arabia

[3] Department of Natural Products and Alternative Medicine, Faculty of Pharmacy,
King Abdulaziz University, Jeddah 21589, Saudi Arabia

[4] Preparatory Year Program, Department of Chemistry, Batterjee Medical College, Jeddah 21442, Saudi Arabia

[5] Department of Pharmacognosy, Faculty of Pharmacy, Assiut University, Assiut 71526, Egypt

* Correspondence: sabrin.ibrahim@bmc.edu.sa; Tel.: +966-581183034

Table S1. Secondary metabolites reported from *Ferula sinkiangensis* (Chemical class, molecular weight and formulae, plant part, and origin).

**Compound Name/Chemical class**	**Mol. Wt.**	**Mol. Formula**	**Plant part**	**Location/Source**	**Ref.**
Bicyclic sesquiterpene coumarins					
Farnesiferol A (**1**)	382.21	$C_{24}H_{30}O_4$	Gum resin	Xinjiang, China	[48]
	-	-	Resin	Baishitun mountain, Yili state, Xinjiang Uygur Autonomous, China	[39]
Colladonin (**2**)	382.21	$C_{24}H_{30}O_4$	Seeds	Yili state, Xinjiang Uygur Autonomous Region, China	[50]
Coladin (**3**)	438.24	$C_{27}H_{34}O_5$	Resin	Xinjiang, China	[25]
Isofeterin (**4**)	440.21	$C_{26}H_{32}O_6$	Roots	Xin jiang Uygur Autonomous Region, China	[30]
Farnesiferone A (**5**)	380.19	$C_{24}H_{28}O_4$	Gum resin	Xinjiang, China	[48]
	-	-	Resin	Xinjiang, China	[25]
	-	-	Resin	Baishitun mountain, Yili state, Xinjiang Uygur Autonomous, China	[39]
Assafoetidnol A (**6**)	398.49	$C_{24}H_{30}O_5$	Resin	Baishitun mountain, Yili state, Xinjiang Uygur Autonomous, China	[39]
Gummosin (**7**)	382.21	$C_{24}H_{30}O_4$	Gum resin	Xinjiang, China	[48]
	-	-	Resin	Baishitun mountain, Yili state, Xinjiang Uygur Autonomous, China	[39]
Polyanthinin (**8**)	424.22	$C_{26}H_{32}O_5$	Gum resin	Xinjiang, China	[48]
	-	-	Resin	Baishitun mountain, Yili state, Xinjiang Uygur Autonomous, China	[39]
Assafoetidnol B (**9**)	456.21	$C_{26}H_{32}O_7$	Resin	Baishitun mountain, Yili state, Xinjiang Uygur Autonomous, China	[39]
Sinkiangenol E (**10**)	440.21	$C_{26}H_{32}O_6$	Resin	Baishitun mountain, Yili state, Xinjiang Uygur Autonomous, China	[39]
Ferukrin (**11**)	400.22	$C_{24}H_{32}O_5$	Gum resin	Xinjiang, China	[48]
	-	-	Resin	Baishitun mountain, Yili state, Xinjiang Uygur Autonomous, China	[39]
(3`S, 5`S, 8`R, 9`S, 10`R)-Kellerin (**12**)	442.23	$C_{26}H_{34}O_6$	Gum resin	Xinjiang, China	[48]

Compound Name/Chemical class	Mol. Wt.	Mol. Formula	Plant part	Location/Source	Ref.
	-	-	Resin	Baishitun mountain, Yili state, Xinjiang Uygur Autonomous, China	[39]
(3`S, 5`S, 8`R,9`S, 10`R)-Deacetylkellerin (13)	400.22	$C_{24}H_{32}O_5$	Gum resin	Xinjiang, China	[48]
	-	-	Resin	Baishitun mountain, Yili state, Xinjiang Uygur Autonomous, China	[39]
Colladocin (14)	442.23	$C_{26}H_{34}O_6$	Resin	Baishitun mountain, Yili state, Xinjiang Uygur Autonomous, China	[39]
Episamarcandin (**15**)	400.22	$C_{24}H_{32}O_5$	Roots	Xinjiang, China	[19]
	-	-	Seeds	Yili state, Xinjiang Uygur Autonomous Region, China	[50]
(5`S, 8`R, 9`S, 10`R)-Ferukrinone (**16**)	398.20	$C_{24}H_{30}O_5$	Gum resin	Xinjiang, China	[48]
	-	-	Resin	Baishitun mountain, Yili state, Xinjiang Uygur Autonomous, China	[39]
Sinkiangenol D (17)	442.23	$C_{26}H_{34}O_6$	Resin	Baishitun mountain, Yili state, Xinjiang Uygur Autonomous, China	[39]
Isosamarcandin (**18**)	380.19	$C_{24}H_{28}O_4$	Roots	Xinjiang, China	[19]
Feselol (**19**)	382.21	$C_{24}H_{30}O_4$	Seeds	Yili state, Xinjiang Uygur Autonomous Region, China	[50]
Ferocaulidin (**20**)	396.19	$C_{24}H_{28}O_5$	Resin	Baishitun mountain, Yili state, Xinjiang Uygur Autonomous, China	[39]
Sinkiangenol A (**21**)	454.19	$C_{26}H_{30}O_7$	Resin	Baishitun mountain, Yili state, Xinjiang Uygur Autonomous, China	[39]
Sinkiangenol B (**22**)	412.18	$C_{24}H_{28}O_6$	Resin	Baishitun mountain, Yili state, Xinjiang Uygur Autonomous, China	[39]
(3`S, 8`R, 9`S, 10`R)-Sinkianol A (**23**)	382.21	$C_{24}H_{30}O_4$	Gum resin	Xinjiang, China	[48]
Lehmannolol (**24**)	384.23	$C_{24}H_{32}O_4$	Roots	Xin jiang Uygur Autonomous Region, China,	[30]
	-	-	Seeds	Yili state, Xinjiang Uygur Autonomous Region, China	[50]
	-	-	Seeds	Yili state, Xinjiang Uygur Autonomous Region, China	[24]

Compound Name/Chemical class	Mol. Wt.	Mol. Formula	Plant part	Location/Source	Ref.
	-	-	Resin	Baishitun mountain, Yili state, Xinjiang Uygur Autonomous, China	[39]
Lehmannolone (**25**)	382.21	$C_{24}H_{30}O_4$	Roots	Xin jiang Uygur Autonomous Region, China,	[30]
	-	-	Seeds	Yili state, Xinjiang Uygur Autonomous Region, China	[50]
	-	-	Resin	Xinjiang, China	[25]
Kamolone (**26**)	382.21	$C_{24}H_{30}O_4$	Resin	Baishitun mountain, Yili state, Xinjiang Uygur Autonomous, China	[39]
	-	-	Resin	Xinjiang, China	[25]
(3`S, 4`S, 5`R, 8`S, 9`S, 10`S)-Kamolol acetate (**27**)	426.24	$C_{26}H_{34}O_5$	Resin	Xinjiang, China	[25]
Ferusingensine H (**28**)	382.21	$C_{24}H_{30}O_4$	Resin	Xinjiang, China	[25]
Monocyclic sesquiterpene coumarins					
Fekrynol (**29**)	384.23	$C_{24}H_{32}O_4$	Roots	Xinjiang, China	[19]
	-	-	Seeds	Yili state, Xinjiang Uygur Autonomous Region, China	[50]
	-	-	Resin	Xinjiang, China	[25]
	-	-	Resin	Baishitun mountain, Yili state, Xinjiang Uygur Autonomous, China	[39]
Fekrynol acetate (**30**)	426.24	$C_{26}H_{34}O_5$	Resin	Xinjiang, China	[25]
	-	-	Resin	Baishitun mountain, Yili state, Xinjiang Uygur Autonomous, China	[39]
(8`S, 9`S, 10`S)-Propionyl fekrynol (**31**)	440.25	$C_{27}H_{36}O_5$	Resin	Xinjiang, China	[25]
Galbanic acid (**32**)	398.20	$C_{24}H_{30}O_5$	Resin	Xinjiang Uygur Autonomous Region, China	[14]
Methyl galbanate (**33**)	412.22	$C_{25}H_{32}O_5$	Gum resin	Xinjiang, China	[48]
	-	-	Resin	Baishitun mountain, Yili state, Xinjiang Uygur Autonomous, China	[39]
Fekolone (**34**)	380.19	$C_{24}H_{28}O_4$	Roots	Xinjiang, China	[19]
	-	-	Seeds	Yili state, Xinjiang Uygur Autonomous Region, China	[50]
Sinkianone (**35**)	382.21	$C_{24}H_{30}O_4$	Roots	Xinjiang Uygur Autonomous Region, China,	[30]

**Compound Name/Chemical class**	**Mol. Wt.**	**Mol. Formula**	**Plant part**	**Location/Source**	**Ref.**
	-	-	Seeds	Yili state, Xinjiang Uygur Autonomous Region, China	[50]
(3`R, 5`R, 10`R)-Sinkianol B (**36**)	400.22	$C_{24}H_{32}O_5$	Gum resin	Xinjiang, China	[48]
Fekrol (**37**)	400.22	$C_{24}H_{32}O_5$	Resin	Baishitun mountain, Yili state, Xinjiang Uygur Autonomous, China	[39]
Ferusingensine G (**38**)	396.19	$C_{24}H_{28}O_5$	Resin	Xinjiang, China	[25]
Farnesiferol B (**39**)	382.21	$C_{24}H_{30}O_4$	Roots	Xinjiang, China	[19]
	-	-	Resin	Xinjiang Uygur Autonomous Region, China	[14]
	-	-	Resin	Baishitun mountain, Yili state, Xinjiang Uygur Autonomous, China	[39]
Farnesiferone B (**40**)	380.18	$C_{24}H_{28}O_4$	Resin	Xinjiang, China	[25]
Farnesiferol C (**41**)	382.21	$C_{24}H_{30}O_4$	Roots	Xinjiang, China	[19]
	-	-	Resin	Xinjiang Uygur Autonomous Region, China	[14]
	-	-	Seeds	Yili state, Xinjiang Uygur Autonomous Region, China	[50]
	-	-	Resin	Baishitun mountain, Yili state, Xinjiang Uygur Autonomous, China	[39]
	-	-	Resin	Xinjiang, China	[25]
Sinkiangenorin F (**42**)	400.22	$C_{24}H_{32}O_5$	Seeds	Yili state, Xinjiang Uygur Autonomous Region, China	[51]
8-O-Acetyl sinkiangenorin F (**43**)	442.23	$C_{25}H_{34}O_6$	Seeds	Yili state, Xinjiang Uygur Autonomous Region, China	[51]
Sinkiangenorin D (**44**)	384.23	$C_{24}H_{32}O_4$	Seeds	Yili state, Xinjiang Uygur Autonomous Region, China	[50]
Sinkiangenorin E (**45**)	400.22	$C_{24}H_{32}O_5$	Seeds	Yili state, Xinjiang Uygur Autonomous Region, China	[52]
Ferusingensine F (**46**)	536.35	$C_{34}H_{48}O_5$	Resin	Xinjiang, China	[25]
Sinkiangenol C (47)	398.20	$C_{24}H_{30}O_5$	Resin	Baishitun mountain, Yili state, Xinjiang Uygur Autonomous, China	[39]

Compound Name/Chemical class	Mol. Wt.	Mol. Formula	Plant part	Location/Source	Ref.
2,3-Dihydro-7-hydroxy-2R*,3S*-dimethyl-2-[4-methyl-5-(4-methyl-2-furyl)-3(E)-pentenyl]-furo[3,2-c]coumarin (**48**)	394.17	$C_{24}H_{26}O_5$	Resin	Baishitun mountain, Yili state, Xinjiang Uygur Autonomous, China	[39]
2,3-Dihydro-7-hydroxy-2R*,3R*-dimethyl-2-[4-methyl-5-(4-methyl-2-furyl)-3(E)-pentenyl]-furo[3,2-c]coumarin (**49**)	394.17	$C_{24}H_{26}O_5$	Resin	Baishitun mountain, Yili state, Xinjiang Uygur Autonomous, China	[39]
2,3-Dihydro-7-hydroxy-2S,3R-dimethyl-2-[4,8-dimethyl-3(E),7-nonadienyl]-furo[3,2-c]coumarin (**50**)	382.21	$C_{24}H_{30}O_4$	Roots	China	[40]
2,3-Dihydro-7-hydroxy-2R,3R-dimethyl-2-[4, 8-dimethyl-3(E),7-nonadienyl]-furo[3,2-c]coumarin (**51**)	382.21	$C_{24}H_{30}O_4$	Roots	China	[40]
Acyclic sesquiterpene coumarins					
Umbelliprenin (**52**)	366.21	$C_{24}H_{30}O_3$	Resin	Xinjiang Uygur Autonomous Region, China	[14]
	-	-	Seeds	Yili state, Xinjiang Uygur Autonomous Region, China	[50] [24]
	-	-	Resin	Xinjiang, China	[25]
	-	-	Resin	Baishitun mountain, Yili state, Xinjiang Uygur Autonomous, China	[39]
10R-Karatavicinol (**53**)	400.22	$C_{24}H_{32}O_5$	Resin	Xinjiang Uygur Autonomous Region, China	[14]
	-	-	Resin	Baishitun mountain, Yili state, Xinjiang Uygur Autonomous, China	[39]
12`-Hydroxy-karatavicinol (**54**)	416.21	$C_{24}H_{32}O_6$	Resin	Baishitun mountain, Yili state, Xinjiang Uygur Autonomous, China	[39]
Karatavicinol A (**55**)	440.25	$C_{27}H_{36}O_5$	Resin	Xinjiang Uygur Autonomous Region, China	[14]
Ferusingensine A (**56**)	426.24	$C_{26}H_{34}O_5$	Resin	Xinjiang, China	[25]
Ferusingensine B (**57**)	384.23	$C_{24}H_{32}O_4$	Resin	Xinjiang, China	[25]
Ferusingensine C (**58**)	550.36	$C_{35}H_{50}O_5$	Resin	Xinjiang, China	[25]
Ferusingensine D (**59**)	536.35	$C_{34}H_{48}O_5$	Resin	Xinjiang, China	[25]
Ferusingensine E (**60**)	382.21	$C_{24}H_{30}O_4$	Resin	Xinjiang, China	[25]
Sesquiterpene chromones					
(+)-Ferulasin 2R, 3R, 10`R (**61**)	398.20	$C_{24}H_{30}O_5$	Roots	Purchased from the Yellow River medicinal materials market of Lanzhou, China	[40]

Compound Name/Chemical class	Mol. Wt.	Mol. Formula	Plant part	Location/Source	Ref.
(-)-Ferulasin 2S, 3S, 10`S (**62**)	398.20	$C_{24}H_{30}O_5$	Roots	Purchased from the Yellow River medicinal materials market of Lanzhou, China	[40]
Acyclic monoterpene coumarins					
Ferusinkin A (**63**)	330.14	$C_{19}H_{22}O_5$	Aerial parts	Yili state, Xinjiang Uygur Autonomous Region, China	[41]
Diversin (**64**)	312.13	$C_{19}H_{20}O_4$	Aerial parts	Yili state, Xinjiang Uygur Autonomous Region, China	[41]
Auraptene (**65**)	312.13	$C_{19}H_{20}O_4$	Aerial parts	Yili state, Xinjiang Uygur Autonomous Region, China	[41]
Coumarins					
Umbelliferone (**66**)	162.03	$C_9H_6O_3$	Roots	Xinjiang, China	[19]
	-	-	Resin	Xinjiang Uygur Autonomous Region, China	[14]
	-	-	Seeds	Xinjiang, China	[27]
Herniarin (**67**)	176.04	$C_{10}H_8O_3$	Aerial parts	Yili state, Xinjiang Uygur Autonomous Region, China	[41]
Sinkiangenol F (**68**)	312.09	$C_{18}H_{16}O_5$	Resin	Baishitun mountain, Yili state, Xinjiang Uygur Autonomous, China	[39]
Sesquiterpene phenylpropanoids					
Sinkiangenone A (**69**)	576.30	$C_{35}H_{44}O_7$	Resin	Yili state, Xinjiang Uygur Autonomous Region, China	[29]
Sinkiangenone B (**70**)	576.30	$C_{35}H_{44}O_7$	Resin	Yili state, Xinjiang Uygur Autonomous Region, China	[29]
Ferulaeone A (**71**)	414.24	$C_{25}H_{34}O_5$	Roots	China	[43]
Ferulaeone G (**72**)	518.30	$C_{33}H_{42}O_5$	Resin	Yili state, Xinjiang Uygur Autonomous Region, China	[29]
Dshamirone (**73**)	356.23	$C_{23}H_{32}O_3$	Roots	China	[40]
1-(2,4-Dihydroxyphenyl)-2-hydroxy-5,9,13 -trimethyl-4(E),8(E),12-tetradecatrien-1-one (**74**)	372.23	$C_{23}H_{32}O_4$	Roots	China	[40]
3S-(2,4-Dihydroxybenzoyl)-4R,5R-dimethyl-5-(4, 8-dimethy-3(E),7(E)-nonadien-1-yl)tetrahydro-2-furanone (**75**)	400.22	$C_{24}H_{32}O_5$	Roots	China	[40]

Compound Name/Chemical class	Mol. Wt.	Mol. Formula	Plant part	Location/Source	Ref.
Fukanedone B (**76**)	400.22	$C_{24}H_{32}O_5$	Roots	China	[40]
Lignans					
Arctigenin (**77**)	372.15	$C_{21}H_{24}O_6$	Seeds	Yili state, Xinjiang Uygur Autonomous Region, China	[17]
	-	-	Seeds	Yili state, Xinjiang Uygur Autonomous Region, China	[24]
Matairesinol (**78**)	420.21	$C_{20}H_{22}O_6$	Seeds	Yili state, Xinjiang Uygur Autonomous Region, China	[17]
Secroisolariciresinol (**79**)	362.17	$C_{20}H_{26}O_6$	Seeds	Yili state, Xinjiang Uygur Autonomous Region, China	[17]
	-	-	Seeds	Xinjiang, China	[27]
Lariciresinol (**80**)	360.15	$C_{20}H_{24}O_6$	Seeds	Yili state, Xinjiang Uygur Autonomous Region, China	[17]
	-	-	Seeds	Yili state, Xinjiang Uygur Autonomous Region, China	[24]
	-	-	Seeds	Xinjiang, China	[27]
(7,8-cis-8,8`-trans)-2-4-Dihydroxyl-3,5-dimethoxy-lariciresinol (**81**)	360.15	$C_{20}H_{24}O_6$	Seeds	Yili state, Xinjiang Uygur Autonomous Region, China	[24]
(+)-Ferulasinkin A (**82**) (7R, 8R, 7`R, 8`R)	362.13	$C_{19}H_{22}O_7$	Resin	Purchased from Huangyadai Pharmaceutical Co., Ltd., Yunnan, China	[35]
(-)-Ferulasinkin A (**83**) (7S, 8S, 7`S, 8`S)	362.13	$C_{19}H_{22}O_7$	Resin	Purchased from Huangyadai Pharmaceutical Co., Ltd., Yunnan, China	[35]
(+)-Ferulasinkin B (**84**) (7S, 8S, 7`R, 8`R)	362.13	$C_{19}H_{22}O_7$	Resin	Purchased from Huangyadai Pharmaceutical Co., Ltd., Yunnan, China	[35]
(-)-Ferulasinkin B (**85**) (7R, 8R, 7`S, 8`S)	362.13	$C_{19}H_{22}O_7$	Resin	Purchased from Huangyadai Pharmaceutical Co., Ltd., Yunnan, China	[35]
(+)-Ferulasinkin C (**86**) (7S, 8S, 7`R, 8`S)	362.13	$C_{19}H_{22}O_7$	Resin	Purchased from Huangyadai Pharmaceutical Co., Ltd., Yunnan, China	[35]
(-)-Ferulasinkin C (**87**) (7R, 8R, 7`S, 8`R)	362.13	$C_{19}H_{22}O_7$	Resin	Purchased from Huangyadai Pharmaceutical Co., Ltd., Yunnan, China	[35]

Compound Name/Chemical class	Mol. Wt.	Mol. Formula	Plant part	Location/Source	Ref.
(+)-Ferulasinkin D (**88**)<br>(7R, 8S, 7`S, 8`S)	362.13	$C_{19}H_{22}O_7$	Resin	Purchased from Huangyadai Pharmaceutical Co., Ltd., Yunnan, China	[35]
(-)-Ferulasinkin D (**89**)<br>(7S, 8R, 7`R, 8`R)	362.13	$C_{19}H_{22}O_7$	Resin	Purchased from Huangyadai Pharmaceutical Co., Ltd., Yunnan, China	[35]
(+)-Sinkianlignan A (**90**)<br>(7``S, 8``R)	518.18	$C_{30}H_{30}O_8$	Resin	Kashgar, Xinjiang Uyghur Autonomous Region, China	[44]
(-)-Sinkianlignan A (**91**)<br>(7``R, 8``S)	518.19	$C_{30}H_{30}O_8$	Resin	Kashgar, Xinjiang Uyghur Autonomous Region, China	[44]
(+)-Sinkianlignan B (**92**)<br>(7S, 8S, 7``S, 8``R)	552.19	$C_{30}H_{32}O_{10}$	Resin	Kashgar, Xinjiang Uyghur Autonomous Region, China	[44]
(-)-Sinkianlignan B (**93**)<br>(7R, 8R, 7``R, 8``S)	552.19	$C_{30}H_{32}O_{10}$	Resin	Kashgar, Xinjiang Uyghur Autonomous Region, China	[44]
(+)-Sinkianlignan C (**94**)<br>(7R, 8R, 9S, 7``R)	524.24	$C_{30}H_{36}O_8$	Resin	Kashgar, Xinjiang Uyghur Autonomous Region, China	[44]
(-)-Sinkianlignan C (**95**)<br>(7S, 8S, 9R, 7``S)	524.24	$C_{30}H_{36}O_8$	Resin	Kashgar, Xinjiang Uyghur Autonomous Region, China	[44]
(+)-Sinkianlignan D (**96**)<br>(7S, 8R, 9``S)	430.16	$C_{23}H_{26}O_8$	Resin	Kashgar, Xinjiang Uyghur Autonomous Region, China	[44]
(-)-Sinkianlignan D (**97**)<br>(7R, 8S, 9``R)	430.16	$C_{23}H_{26}O_8$	Resin	Kashgar, Xinjiang Uyghur Autonomous Region, China	[44]
(+)-Sinkianlignan E (**98**)<br>(7S, 85)	358.17	$C_{21}H_{26}O_5$	Resin	Kashgar, Xinjiang Uyghur Autonomous Region, China	[44]
(-)-Sinkianlignan E (**99**)<br>(7R, 8R)	358.17	$C_{21}H_{26}O_5$	Resin	Kashgar, Xinjiang Uyghur Autonomous Region, China	[44]
(+)-Sinkianlignan F (**100**)<br>(7R, 85)	358.17	$C_{21}H_{26}O_5$	Resin	Kashgar, Xinjiang Uyghur Autonomous Region, China	[44]
(-)-Sinkianlignan F (**101**)<br>(7S, 8R)	358.17	$C_{21}H_{26}O_5$	Resin	Kashgar, Xinjiang Uyghur Autonomous Region, China	[44]
(+)-Sinkianlignan G (**102**)<br>(7R, 8S, 7`R, 8`S)	376.15	$C_{20}H_{24}O_7$	Resin	Kashgar, Xinjiang Uyghur Autonomous Region, China	[18]

Compound Name/Chemical class	Mol. Wt.	Mol. Formula	Plant part	Location/Source	Ref.
(-)-Sinkianlignan G (**103**) (7S, 8R, 7`S, 8`R)	376.15	$C_{20}H_{24}O_7$	Resin	Kashgar, Xinjiang Uyghur Autonomous Region, China	[18]
(+)-Sinkianlignan H (**104**) (7S, 8R, 7`R, 8`R)	376.15	$C_{20}H_{24}O_7$	Resin	Kashgar, Xinjiang Uyghur Autonomous Region, China	[18]
(-)-Sinkianlignan H (**105**) (7R, 8S, 7`S, 8`S)	376.15	$C_{20}H_{24}O_7$	Resin	Kashgar, Xinjiang Uyghur Autonomous Region, China	[18]
(+)-Sinkianlignan I (**106**) (7R, 8R, 7`S, 8`S)	376.15	$C_{20}H_{24}O_7$	Resin	Kashgar, Xinjiang Uyghur Autonomous Region, China	[18]
(-)-Sinkianlignan I (**107**) (7S, 8S, 7`R, 8`R)	376.15	$C_{20}H_{24}O_7$	Resin	Kashgar, Xinjiang Uyghur Autonomous Region, China	[18]
(+)-Sinkianlignan J (**108**) (7R, 8R, 7`R, 8`R)	376.15	$C_{20}H_{24}O_7$	Resin	Kashgar, Xinjiang Uyghur Autonomous Region, China	[18]
(-)-Sinkianlignan J (**109**) (7S, 8S, 7`S, 8`S)	376.15	$C_{20}H_{24}O_7$	Resin	Kashgar, Xinjiang Uyghur Autonomous Region, China	[18]
(+)-Sinkianlignan K (**110**) (7R, 8S, 7`S, 8`R)	376.15	$C_{20}H_{24}O_7$	Resin	Kashgar, Xinjiang Uyghur Autonomous Region, China	[18]
(-)-Sinkianlignan K (**111**) (7S, 8S, 7`R, 8`S)	376.15	$C_{20}H_{24}O_7$	Resin	Kashgar, Xinjiang Uyghur Autonomous Region, China	[18]
Neoarctin A (**112**)	742.29	$C_{42}H_{46}O_{12}$	Seeds	Xinjiang, China	[27]
Pinoresinol (**113**)	358.14	$C_{20}H_{22}O_6$	Seeds	Xinjiang, China	[27]
Arctiin (**114**)	534.21	$C_{27}H_{34}O_{11}$	Seeds	Xinjiang, China	[27]
Monoterpenes					
Carene (**115**)	136.12	$C_{10}H_{16}$	Oleo-gum resin	Xinjiang, China	[8]
Camphene (**116**)	136.12	$C_{10}H_{16}$	Oleo-gum resin	Xinjiang, China	[8]
β-Pinene (**117**)	136.12	$C_{10}H_{16}$	Oleo-gum resin	Xinjiang, China	[8]
α- Pinene (**118**)	136.12	$C_{10}H_{16}$	Oleo-gum resin	Xinjiang, China	[8]

Compound Name/Chemical class	Mol. Wt.	Mol. Formula	Plant part	Location/Source	Ref.
p-Cymene (**119**)	134.10	$C_{10}H_{14}$	Oleo-gum resin	Xinjiang, China	[8]
Sesquiterpenes					
Nerolidol (**120**)	222.19	$C_{15}H_{26}O$	Aerial parts	Yili state, Xinjiang Uygur Autonomous Region, China	[41]
(3S,E)-Nerolidol (**121**)	222.19	$C_{15}H_{26}O$	Roots	China	[40]
Guaiol (**122**)	222.19	$C_{15}H_{26}O$	Aerial parts	Yili state, Xinjiang Uygur Autonomous Region, China	[41]
	-	-	Roots	China	[40]
Brevifoliol (**123**)	556.26	$C_{31}H_{40}O_9$	Seeds	Xinjiang, China	[27]
Fercoperol (**124**)	270.18	$C_{15}H_{26}O_4$	Roots	China	[40]
iso-Fercoperol (**125**)	270.18	$C_{15}H_{26}O_4$	Roots	China	[40]
Sulfanes					
1-(sec-Butyl)-2-methyldisulfane (**126**)	136.03	$C_5H_{12}S_2$	Oleo-gum resin	Xinjiang, China	[8]
1-(sec-Butyl)-2-ethyldisulfane (**127**)	150.05	$C_6H_{14}S_2$	Oleo-gum resin	Xinjiang, China	[8]
1-(sec-Butyl)-2-vinyldisulfane (**128**)	148.03	$C_6H_{12}S_2$	Oleo-gum resin	Xinjiang, China	[8]
1-Butyl-2-ethyldisulfane (**129**)	150.05	$C_6H_{14}S_2$	Oleo-gum resin	Xinjiang, China	[8]
1,3-Dimethyltrisulfane (**130**)	125.96	$C_2H_6S_3$	Oleo-gum resin	Xinjiang, China	[8]
1-(sec-Butyl)-2-isopropyldisulfane (**131**)	164.06	$C_7H_{16}S_2$	Oleo-gum resin	Xinjiang, China	[8]
1,2-di-sec-Butyldisulfane (**132**)	178.08	$C_8H_{18}S_2$	Oleo-gum resin	Xinjiang, China	[8]
(Z)-1-(sec-Butyl)-2-(prop-1-en-1-yl)disulfane (**133**)	162.05	$C_7H_{14}S_2$	Oleo-gum resin	Xinjiang, China	[8]
	-	-	Resin	Xin Jiang, China	[60]

Compound Name/Chemical class	Mol. Wt.	Mol. Formula	Plant part	Location/Source	Ref.
(Z)-1-(But-1-en-1-yl)-2-(sec-butyl)disulfane (**134**)	176.06	$C_8H_{16}S_2$	Oleo-gum resin	Xinjiang, China	[8]
(E)-1-(But-1-en-1-yl)-2-(sec-butyl)disulfane (**135**)	176.06	$C_8H_{16}S_2$	Oleo-gum resin	Xinjiang, China	[8]
	-	-	Resin	Xin Jiang, China	[60]
1,3-di-sec-Butyltrisulfane (**136**)	210.05	$C_8H_{18}S_3$	Oleo-gum resin	Xinjiang, China	[8]
(E)-1-(sec-Butyl)-3-(prop-1-en-1-yl)trisulfane (**137**)	194.02	$C_7H_{14}S_3$	Oleo-gum resin	Xinjiang, China	[8]
1-(2-(Isopropylthio)ethyl)-2-methyldisulfane (**138**)	182.02	$C_6H_{14}S_3$	Oleo-gum resin	Xinjiang, China	[8]
1-(3-(Isopropylthio)propyl)-2-methyldisulfane (**139**)	196.04	$C_7H_{16}S_3$	Oleo-gum resin	Xinjiang, China	[8]
(Z)-1-(3-(Isopropylthio)prop-1-en-1-yl)-2-methyldisulfane (**140**)	194.02	$C_7H_{14}S_3$	Oleo-gum resin	Xinjiang, China	[8]
(E)-1-(2-(Isopropylthio)vinyl)-2-methyldisulfane (**141**)	180.01	$C_6H_{12}S_3$	Oleo-gum resin	Xinjiang, China	[8]
1-(2-(sec-Butylthio)ethyl)-2-methyldisulfane (**142**)	196.04	$C_7H_{16}S_3$	Oleo-gum resin	Xinjiang, China	[8]
1-(3-(sec-Butylthio)propyl)-2-methyldisulfane (**143**)	210.05	$C_8H_{18}S_3$	Oleo-gum resin	Xinjiang, China	[8]
(E)-1-Methyl-2-(2-(propylthio)vinyl)disulfane (**144**)	180.01	$C_6H_{12}S_3$	Oleo-gum resin	Xinjiang, China	[8]
(Z)-1-(3-(sec-Butylthio)prop-1-en-1-yl)-2-methyldisulfane (**145**)	208.04	$C_8H_{16}S_3$	Oleo-gum resin	Xinjiang, China	[8]
(Z)-1-(2-(sec-butylthio)vinyl)-2-methyldisulfane (**146**)	194.02	$C_7H_{14}S_3$	Oleo-gum resin	Xinjiang, China	[8]
1-Methyl-2-((E)-2-(((E)-prop-1-en-1-yl) disulfanyl)vinyl)disulfane (**147**)	209.96	$C_6H_{10}S_4$	Oleo-gum resin	Xinjiang, China	[8]
Steroids					

Compound Name/Chemical class	Mol. Wt.	Mol. Formula	Plant part	Location/Source	Ref.
Sinkiangenrin A (**148**)	420.21	$C_{23}H_{32}O_7$	Seeds	Yili state, Xinjiang Uygur Autonomous Region, China	[17]
Sinkiangenrin B (**149**)	418.19	$C_{23}H_{30}O_7$	Seeds	Yili state, Xinjiang Uygur Autonomous Region, China	[17]
β-Sitosterol (**150**)	414.38	$C_{29}H_{50}O$	Seeds	Xinjiang, China	[27]
Stigmasterol (**151**)	412.37	$C_{29}H_{48}O$	Seeds	Xinjiang, China	[27]
Daucosterol (**152**)	576.43	$C_{35}H_{60}O_6$	Seeds	Xinjiang, China	[27]
Stigmasterol-3-O-β-D-glucoside (**153**)	574.42	$C_{35}H_{58}O_6$	Seeds	Xinjiang, China	[27]
Phenolic compounds					
Quercetin (**154**)	302.04	$C_{15}H_{10}O_7$	Seeds	Yili state, Xinjiang Uygur Autonomous Region, China	[24]
Isoquercitrin (**155**)	464.09	$C_{21}H_{20}O_{12}$	Seeds	Xinjiang, China	[27]
Rutin (**156**)	610.15	$C_{27}H_{30}O_{16}$	Seeds	Xinjiang, China	[27]
Luteolin-7-O-β-D-glucuronide (**157**)	462.07	$C_{21}H_{18}O_{12}$	Seeds	Xinjiang, China	[27]
Ferulic acid (**158**)	194.05	$C_{10}H_{10}O_4$	Seeds	Xinjiang, China	[27]
			Resin	Yili state, Xinjiang Uygur Autonomous Region, China	[29]
	-	-	Aerial parts	Yili state, Xinjiang Uygur Autonomous Region, China	[41]
	-	-	Resin	Kashgar, Xinjiang Uyghur Autonomous Region, China	[18]
Caffeic acid (**159**)	180.04	$C_9H_8O_4$	Seeds	Xinjiang, China	[27]
Chlorogenic acid (**160**)	354.09	$C_{16}H_{18}O_9$	Seeds	Xinjiang, China	[27]
Methyl chlorogenate (**161**)	368.11	$C_{17}H_{20}O_9$	Seeds	Xinjiang, China	[27]
1,5-di-O-Caffeoylquinic (**162**)	516.12	$C_{25}H_{24}O_{12}$	Seeds	Xinjiang, China	[27]
P-Hydroxycinnamic acid (**163**)	164.04	$C_9H_8O_3$	Resin	Yili state, Xinjiang Uygur Autonomous Region, China	[29]
3`,4`-Dimethoxy cinnamyl isovalent (**164**)	278.15	$C_{16}H_{22}O_4$	Resin	Yili state, Xinjiang Uygur Autonomous Region, China	[29]
Vanillic acid (**165**)	168.04	$C_8H_8O_4$	Seeds	Xinjiang, China	[27]

Compound Name/Chemical class	Mol. Wt.	Mol. Formula	Plant part	Location/Source	Ref.
Vanillin (**166**)	152.04	$C_8H_8O_3$	Resin	Yili state, Xinjiang Uygur Autonomous Region, China	[29]
Coniferaldehyde = Ferulaldehyde (**167**)	178.06	$C_{10}H_{10}O_3$	Resin	Yili state, Xinjiang Uygur Autonomous Region, China	[29]
	-	-	Resin	Kashgar, Xinjiang Uyghur Autonomous Region, China	[18]
(2E)-3-(4-Ethoxy-3-methoxyphenyl)prop-2-en-1-ol (**168**)	208.10	$C_{12}H_{16}O_3$	Resin	Yili state, Xinjiang Uygur Autonomous Region, China	[29]
(7S,8R)-Guaiacyl glycerol (**169**)	214.08	$C_{10}H_{14}O_5$	Resin	Yili state, Xinjiang Uygur Autonomous Region, China	[29]
(7S,8S)-Guaiacyl glycerol (**170**)	214.08	$C_{10}H_{14}O_5$	Resin	Yili state, Xinjiang Uygur Autonomous Region, China	[29]
3`,4`-dimethoxycinnamyl alcohol (**171**)	194.09	$C_{11}H_{14}O_3$	Resin	Kashgar, Xinjiang Uyghur Autonomous Region, China	[18]
4-[(E)-3-ethoxyprop-1-enyl]-2-methoxy phenol (**172**)	208.10	$C_{12}H_{16}O_3$	Resin	Kashgar, Xinjiang Uyghur Autonomous Region, China	[18]
Brachangobinan E (**173**)	312.15	$C_{16}H_{24}O_6$	Resin	Kashgar, Xinjiang Uyghur Autonomous Region, China	[18]
4,4`-Dihydroxy-3,3`-dimethoxy-trans-stilbene (**174**)	272.10	$C_{16}H_{16}O_4$	Resin	Yili state, Xinjiang Uygur Autonomous Region, China	[29]
3,3`-Dimethoxy-4,4'-dioxychalcone (**175**)	300.09	$C_{17}H_{16}O_5$	Resin	Kashgar, Xinjiang Uyghur Autonomous Region, China	[18]
Ferulagenol A (**176**)	290.11	$C_{16}H_{18}O_5$	Resin	Kashgar, Xinjiang Uyghur Autonomous Region, China	[18]
4-Hyroxy-3-methoxyphnylerulate (**177**)	316.09	$C_{17}H_{16}O_6$	Resin	Kashgar, Xinjiang Uyghur Autonomous Region, China	[18]
Macrathoin F (**178**)	530.14	$C_{26}H_{26}O_{12}$	Seeds	Yili state, Xinjiang Uygur Autonomous Region, China	[24]
	-	-	Seeds	Xinjiang, China	[27]
Sinkiangenone C (**179**)	390.13	$C_{20}H_{22}O_8$	Resin	Yili state, Xinjiang Uygur Autonomous Region, China	[29]

Compound Name/Chemical class	Mol. Wt.	Mol. Formula	Plant part	Location/Source	Ref.
Sinkiangenone D (**180**)	390.13	$C_{20}H_{22}O_8$	Resin	Yili state, Xinjiang Uygur Autonomous Region, China	[29]
3-(3,4-Dimethoxyphenyl)-2-propen-1-yl ester (**181**)	370.14	$C_{21}H_{22}O_6$	Resin	Yili state, Xinjiang Uygur Autonomous Region, China	[29]
Other metabolites					
Sinkiangensis A (**182**)	282.14	$C_{15}H_{22}O_5$	Resin	Yining, Xinjiang, China	[46]
Sinkiangensis B (**183**)	324.15	$C_{17}H_{24}O_6$	Resin	Yining, Xinjiang, China	[46]
Sinkiangensis C (**184**)	240.09	$C_{12}H_{16}O_5$	Resin	Yining, Xinjiang, China	[46]
5-Hydroxymethyl fufural (**185**)	126.03	$C_6H_6O_3$	Seeds	Xinjiang, China	[27]
Variecolorquinone A (**186**)	402.09	$C_{20}H_{18}O_9$	Seeds	Xinjiang, China	[27]
1-(3-Ethylphenyl)-1,2-ethanediol (**187**)	166.09	$C_{10}H_{14}O_2$	Seeds	Xinjiang, China	[27]
Sucrose (**188**)	342.11	$C_{12}H_{22}O_{11}$	Seeds	Xinjiang, China	[27]
Inosine (**189**)	268.08	$C_{10}H_{12}N_4O_5$	Seeds	Xinjiang, China	[27]
Uridine (**190**)	244.06	$C_9H_{12}N_2O_6$	Seeds	Xinjiang, China	[27]
(R)-2`-hydroxy-N-[(2S,3S,4R)-1,3,4-trihydroxyhexacosan-2-yl] nonadanamide (**191**)	585.53	$C_{35}H_{71}NO_5$	Seeds	Xinjiang, China	[27]
Sinkiangenrin C (**192**)	338.15	$C_{14}H_{26}O_9$	Seeds	Yili state, Xinjiang Uygur Autonomous Region, China	[17]
Butanedioic acid (**193**)	118.02	$C_4H_6O_4$	Seeds	Xinjiang, China	[27]
n-Hexacosanol (**194**)	382.41	$C_{26}H_{54}O$	Seeds	Xinjiang, China	[27]

References

[8] Zhi-da, M.; Qi-fi, M.; Mizuno, M.; Tanaka, T.; Iinuma, M. Polysulfanes in the volatile oils of *Ferula* species. *Planta Med*. **1987**, *53*, 300-302.

[13] Sattar, Z.; Iranshahi, M. Phytochemistry and pharmacology of *Ferula hermonis* Boiss.–A review. Drug Res. **2017**, *67*, 437-446.

[14] Teng, L.; Ma, G.Z.; Li, L.; Ma, L.Y.; Xu, X.Q. Karatavicinol a, a new anti-ulcer sesquiterpene coumarin from *Ferula Sinkiangensis*. *Chem. Nat. Compd*. **2013**, *49*, 606-609.

[17] Li, G.; Li, X.; Cao, L.; Shen, L.; Zhu, J.; Zhang, J.; Wang, J.; Zhang, L.; Si, J. Steroidal esters from *Ferula sinkiangensis*. *Fitoterapia* **2014**, *97*, 247-252.

[18] Yan, Y.; Bao, X.; Li, Y.; Li, Q.; Cheng, Y.; Jiao, Y.; Zhang, H. Racemic Norneolignans from the resin of *Ferula sinkiangensis* and their Cox-2 inhibitory activity. *Fitoterapia* **2023**, *164*, 105341.

[19] Yang, J.; Jing, S.; Li, Z.; Qin, H. Chemical constituents from roots of *Ferula sinkiangensis*. *Zhongguo Zhong Yao Za Zhi* **2007**, *32*, 2382-2384.

[24] Zhang, L.; Si, J.; Li, G.; Li, X.; Zhang, L.; Gao, L.; Huo, X.; Liu, D.; Sun, X.; Cao, L. Umbelliprenin and lariciresinol isolated from a long-term-used herb medicine *Ferula sinkiangensis* induce apoptosis and G0/G1 arresting in gastric cancer cells. RSC Adv. **2015**, *5*, 91006-91017.

[25] Guo, T.; Zhou, D.; Yang, Y.; Zhang, X.; Chen, G.; Lin, B.; Sun, Y.; Ni, H.; Liu, J.; Hou, Y. Bioactive sesquiterpene coumarins from the resin of *Ferula sinkiangensis* targeted on over-activation of microglia. *Bioorg. Chem*. **2020**, *104*, 104338.

[27] Li, G. Chemical constituents from seeds of *Ferula sinkiangensis*. *Chin. Tradit. Herb. Drugs*. **2015**, 1730-1736.

[29] Wang, J.; Gao, Y.; Wang, H.; Chen, L.; Cao, L.; Xu, J.; Li, X.; Zhao, Y.; Zhu, J.; Si, J. Apoptosis induction and cell cycle arrest induced by sinkiangenone B, a novel phenylpropanoid derivative from the resin of *Ferula sinkiangensis* KM Shen. *RSC Adv.* **2018**, *8*, 4093-4103.

[30] Yang, J.; An, Z.; Li, Z.; Jing, S.; Qina, H. Sesquiterpene coumarins from the roots of *Ferula sinkiangensis* and *Ferula teterrima*. *Chem. Pharm. Bull*. **2006**, *54*, 1595-1598.

[35] Li, Y.; Yang, B.; Zheng, S.; Cheng, Y.; Cui, H. Racemic norlignans as diastereoisomers from *Ferula sinkiangensis* resins with antitumor and wound-healing promotion activities. *Molecules* **2022**, *27*, 3907.

[39] Wang, J.; Wang, H.; Zhang, M.; Li, X.; Zhao, Y.; Chen, G.; Si, J.; Jiang, L. Sesquiterpene coumarins from *Ferula sinkiangensis* KM Shen and their cytotoxic activities. *Phytochemistry* **2020**, *180*, 112531.

[40] Wang, J.; Meng, X.; Zheng, Y.; Sang, C.; Wang, W.; Ma, J.; Zhao, Y.; Yang, J. (±)-Ferulasin, unusual sesquiterpene chromones from *Ferula sinkiangensis*. *Tetrahedron* **2022**, *122*, 132953.

[41] Liu, T.; Wang, L.; Zhang, L.; Jiang, H.; Zhang, Y.; Mao, L. Insecticidal, cytotoxic and anti-phytopathogenic fungal activities of chemical constituents from the aerial parts of *Ferula sinkiangensis*. *Nat. Prod. Res.* **2020**, *34*, 1430-1436.

[43] Wang, J.; Zhao, Y.; Qiang, Y. Chemical constituents from *Ferula sinkiangensis* and their chemotaxonomic significance. *Biochem. Syst. Ecol.* **2022**, *105*, 104519.

[44] Li, Q.; Li, J.; Bao, X.; Zhang, S.; Luo, Q.; Li, K.; Jiao, Y.; Cheng, Y.; Yan, Y. Unusual sesquilignans with anti-inflammatory activities from the resin of *Ferula sinkiangensis*. *Bioorg. Chem*. **2022**, *127*, 105986.

[46] Yi, X.; Li, Z.; Zheng, Q.; Sang, R.; Li, H.; Gao, G.; Qin, Q.; Zhu, N. Three new tetrahydrobenzofuran derivatives from *Ferula sinkiangensis* KM shen and their cytotoxic activities. *Nat. Prod. Res.* **2022**, 1-5. doi: 10.1080/14786419.2022.2075361.

[48] Xing, Y.; Li, N.; Zhou, D.; Chen, G.; Jiao, K.; Wang, W.; Si, Y.; Hou, Y. Sesquiterpene coumarins from *Ferula sinkiangensis* act as neuroinflammation inhibitors. *Planta Med.* **2017**, *83*, 135-142.

[50] Li, G.; Wang, J.; Li, X.; Cao, L.; Gao, L.; Lv, N.; Si, J. An unusual sesquiterpene coumarin from the seeds of *Ferula sinkiangensis*. *J. Asian Nat. Prod. Res.* **2016**, *18*, 891-896.

[51] Li, G.; Li, X.; Cao, L.; Zhang, L.; Shen, L.; Zhu, J.; Wang, J.; Si, J. Sesquiterpene coumarins from seeds of *Ferula sinkiangensis*. *Fitoterapia* **2015**, *103*, 222-226.

[52] Li, G.; Wang, J.; Li, X.; Cao, L.; Lv, N.; Chen, G.; Zhu, J.; Si, J. Two New sesquiterpene coumarins from the seeds of *Ferula sinkiangensis*. *Phytochem. Lett*. **2015**, *13*, 123-126.
[60] Ye, B.; Wang, S.; Zhang, L. Studies on the detoxification effects and acute toxicity of a mixture of cis-sec-butyl-1-propoenyl disulphide and trans-sec-butyl-1-propoenyl disulphide isolated from crude essential oil of *Ferula sinkiangensis* KM Shen, a Chinese traditional herbal medicine. *Nat. Prod. Res.* **2011**, *25*, 1161-1170.
